# Synthesis of Adenine Nucleosides with a Reactive (*β*-Iodovinyl)sulfone or (*β*-Keto)sulfone Group at the C2 Position and Their Polymerase-Catalyzed Incorporation into DNA

**DOI:** 10.3390/molecules30061358

**Published:** 2025-03-18

**Authors:** A. Hasan Howlader, Richard Fernandez, Pawlos S. Tsegay, Yuan Liu, Stanislaw F. Wnuk

**Affiliations:** 1 Department of Chemistry and Biochemistry, Florida International University, Miami, FL 33199, USA; hasanchek56@gmail.com (A.H.H.); yualiu@fiu.edu (Y.L.); 2 Biomolecular Sciences Institute, Florida International University, Miami, FL 33199, USA

**Keywords:** nucleosides, purines, halosulfonylation of alkynes, (*β*-halovinyl)sulfones, (*β*-keto)sulfones, *β*-sulfonylvinylamines, phosphorylation, DNA polymerases, adenosine-2-carboxylic acid

## Abstract

Iodosulfonylation of an ethynyl group at the C2 position of 2′-deoxyadenosine or adenosine with TsI provides (*E*)-2-(*β*-iodovinyl)sulfones. The latter undergo nucleophilic substitution with amines via an addition–elimination to give *β*-sulfonylvinylamines (enamines). Acid-catalyzed hydrolysis of the *β*-sulfonylvinylamines provides 2-(*β*-keto)sulfones, mechanistically different probes that react with alkyl halides, resulting in α-alkylation. Adenine nucleosides with a β-ketosulfone group at C2, during conversion to their 5′-triphosphate form, undergo an unexpected conversion to 2-carboxylic acid nucleotides. The 5′-triphosphate of 2′-deoxyadenosine-2-carboxylic acid was incorporated by a human DNA polymerase into a one-nucleotide gap DNA substrate.

## 1. Introduction

Modification of purine nucleobases provides a strategy for the development of anticancer and antiviral nucleoside therapeutics [1,2]. Methods for the synthesis of C2-substituted purine nucleosides, although, in general, more challenging than those for the C8-substituted counterparts, can now be achieved quite efficiently using several strategies which include (i) nucleophilic aromatic substitutions [1], (ii) diazoniation/dediazoniation sequences [3], (iii) lithiation followed by captures of electrophiles [4], and most effectively by (iv) transition metal-catalyzed cross-coupling reactions [5,6,7], which include methods for direct C-H activation [8], and recently reported (v) regioselective homolytic C2-H borylation through photocatalysis or thermal activation [9].

Purine nucleosides with C2 substitution are a frequently occurring motif in approved drugs and bioactive adenine derivatives [9,10,11,12,13,14], including adenosine receptor ligands [15,16]. Matsuda and Jacobsen groups described C2-substituted adenine nucleosides as adenosine A2 receptor agonists [17], and A2A adenosine receptor antagonists [18], respectively. dATP modified with a reactive formyl group at C2 is used for crosslinking with peptides and proteins, as well as for fluorescent labelling [19]. 2-methyladenosine (m^2^A) promotes protein translation by facilitating the decoding of tandem m^2^A-tRNA-dependent codons [20], and the introduction of hydrophilic PEG substituents at the C2 position of adenine improves water solubility without affecting binding properties [21].

The Hocek group has reported C2-substituted dATP derivatives bearing Cl, NH_2_, CH_3_, vinyl, ethynyl, and phenyl substituents to study the effect of substituents on their polymerase-catalyzed DNA incorporation [22]. They found that all of dATP derivatives were good substrates in primer-extension experiments, except for the bulky 2-phenyl-dATP. Among them, the vinyl-modified DNA reacted with the thiol 4-methylmercaptocoumarin, and ethynyl-modified DNA underwent reaction with Cy3 azide to enzymatically label the minor grove via nucleobase modification. Previously, 2-chloroadenine [23] and 2,6-diaminopurine [24,25] dNTPs were the only reported minor-groove base-modified nucleotides as substrates for DNA polymerases. The Hocek group also reported 2-alkylamino-2′-deoxyadenosine triphosphates, which incorporated only one modified nucleotide into the primer by Terminator DNA polymerase [26]. The allylamino-substituted DNA was utilized for the thiol–ene addition, while the propargylamino-DNA reacted with an azide to label the DNA with a fluorescent dye in the minor groove. Also, 2-substituted 2′-deoxyinosine triphosphates containing a CH_3_ or vinyl group were shown to be good substrates for DNA polymerase and for the enzymatic synthesis of minor-groove base-modified DNA [27].

We reported 5-(1-halo-2-tosylvinyl) pyrimidine nucleosides, their reactions with nucleophiles, and their crosslinking with amino acids via an addition–elimination reaction [28,29,30], as well as the reactivity of the corresponding 5-(2-tosylacetyl) analogues with electrophiles and their polymerase-catalyzed incorporation into DNA [30]. Recently, we developed purine nucleosides modified at C8 with (*β*-halovinyl)sulfone, and (*β*-keto)sulfone probes and determined their DNA incorporation by human or *Escherichia coli* DNA–polymerase-catalyzed reactions [31]. Herein, we describe adenine nucleosides modified at C2 with reactive (*β*-halovinyl)sulfone or *β*-ketosulfone group, as well as the DNA incorporation of 2′-deoxyadenosine-2-carboxylic acid triphosphate by a DNA–polymerase-catalyzed reaction.

## 2. Results and Discussions

**Scheme 1:**
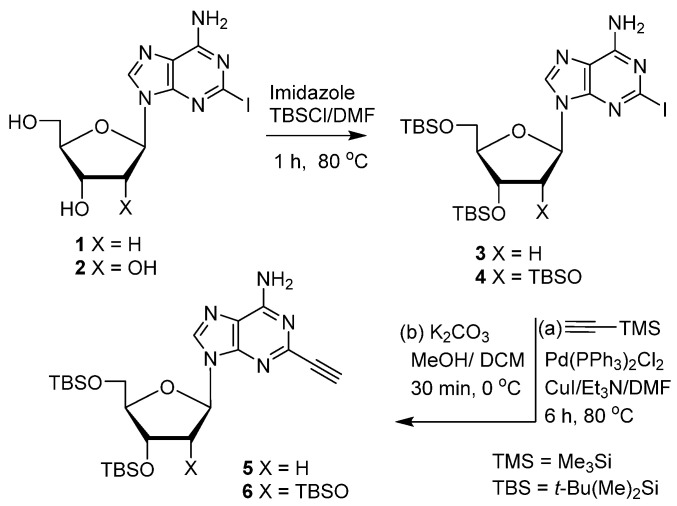
Synthesis of 2-ethynyladenine nucleoside substrates.

The substrates 2-iodo-2′-deoxyadenosine **1** [32] and 2-iodoadenosine **2** [33] (Scheme 1) were synthesized as reported. Standard *tert*-butyldimethylsilyl (TBS) protection of **1** and **2,** followed by the Sonogashira coupling of the resulting **3** [34] and **4** [5], and selective removal of trimethylsilyl (TMS) protection from the 2-ethynyl group, gave the precursors 3′,5′-di-*O*-TBS-2-ethynyl-2′-deoxyadenosine **5** and 2′,3′,5′-tri-*O*-TBS-2-ethynyladenosine **6**.

We initially attempted iodosulfonylation [35,36] of **5** with the I_2_/TsNa/NaOAc [37] (MeCN/80 °C) protocol, which was successful for the iodovinylsulfonylation of 8-alkynyladenine nucleosides [31]. However, the isolated product was identified as 2-(iodoethynyl)-2′-deoxyadenosine **7** (80%; Table 1, entry 1). An analogous reaction in the absence of NaOAc in H_2_O or H_2_O/MeCN mixture also provided **7** but with lower yields (entries 2 and 3). It is noteworthy that such iodination of the terminal alkyne was also observed with 8-ethynyl-2′-deoxyguanosine but not with the adenine counterpart [31]. Also, iodosulfonylation of **5** with TsNHNH_2_/KI/benzoyl peroxide failed to give the expected product **8**, resulting only in partial removal of the TBS-protecting groups (entry 4), although these conditions were successful with 8-alkynylguanine nucleosides [31]. The use of CuI as a source of iodide (entry 5) and application of other methods [38,39,40,41] were also not successful (entries 6 and 7). Furthermore, treatment of **5** with freshly prepared TsI [42] in THF, although it effected iodosulfonylation, resulted mainly in the cleavage of the glycosidic bond (entry 8). However, a similar iodosulfonylation of **5** with TsI (2 equiv.) in the presence of NaOAc (3 equiv.) afforded the expected iodovinylsulfone **8** as a single *E*-isomer (81%; entry 9). Analogous treatment of the adenosine substrate **6** gave **9** in even higher yield (*E*; 92%, entry 10). This condition has a general character and was also successful for the iodosulfonylation of 8-alkynyl nucleosides. For example, treatment of 3′,5′-tri-*O*-(tert-butyldimethylsilyl)-8-ethynyl-2′-deoxyadenosine with TsI in the presence of NaOAc gave (*E*)-3′,5′-tri-O-(tert-butyldimethylsilyl)-8-(1-iodo-2-tosylvinyl)-2′-deoxyadenosine [31] in 78% yield.

**Scheme 2:**
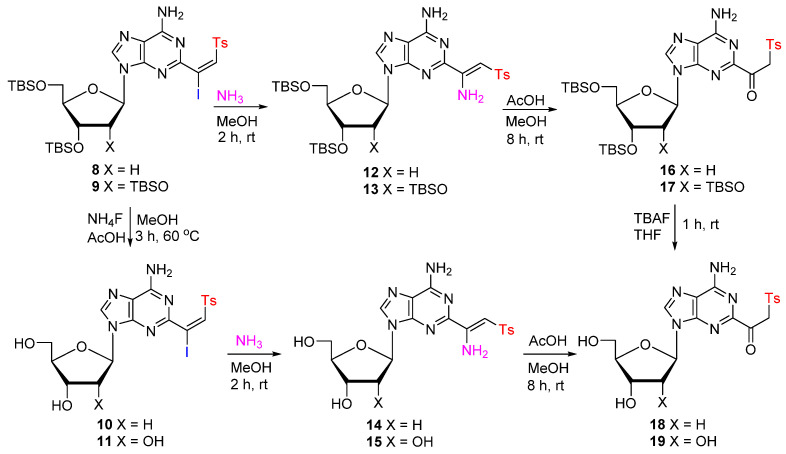
Synthesis of C2-modified *β*-ketosulfone from (*β*-iodo)vinylsulfone.

Treatment of **8** or **9** with NH_4_F/AcOH in MeOH at 60 °C for 6 h gave unprotected 2-(*β*-iodovinyl)sulfone **10** (78%) or **11** (80%) as single *E*-isomers (Scheme 2). Subsequent treatment of **10** or **11** with NH_3_/MeOH (rt, 1 h) provided the corresponding (*Z*)-*β*-aminovinylsulfones **14** (70%) or **15** (70%). The *Z* stereochemistry is believed to be the result of hydrogen bonding between the hydrogen from the NH_2_ group and the oxygen from a sulfonyl group [43,44]. The protected (*Z*)-*β*-aminovinylsulfones **12** and **13** were analogously prepared from **8** or **9**. Reaction of **14** or **15** with AcOH (aq) in MeOH (rt, 24 h) effected efficient conversion to the *β*-ketosulfones [37,45] **18** (84%) or **19** (82%). Similarly, TBS-protected *β*-ketosulfones **16** (86%) and **17** (85%) were prepared from **12** or **13**. Subsequent removal of the silyl protection group with tetrabutylammonium fluoride (TBAF) gave unprotected *β*-ketosulfones **18** (87%) and **19** (88%).

**Scheme 3:**
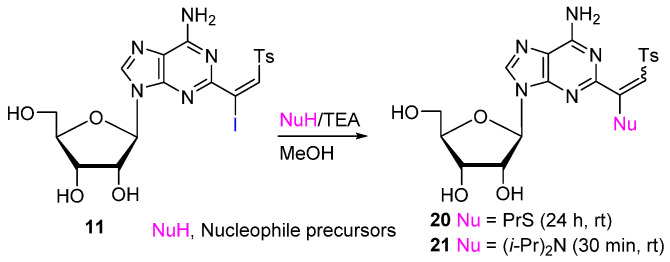
Reactions of 2-(*β*-iodovinyl)sulfone of adenosine with nucleophiles.

As expected [1,2,9], the C2-modified (at electron-deficient pyrimidine ring) adenine nucleosides have different physical, chemical, and spectroscopical properties as compared to C8-modified (at electron-excessive imidazole ring) counterparts [31]. For example, C2 aminovinylsulfones **12**–**15** partially convert into corresponding β-ketosulfones **16**–**19** during silica column purification, while C8 aminovinylsulfones [31] are stable on the silica column. Polarity and Rf value for TLC (Thin-Layer Chromatography) are also quite different, probably due to different internal hydrogen bonding. Especially, the β-ketosulfones at the C2 position of purine rings **16**–**19** are much polar than the corresponding C8 analogues.

(*β*-halovinyl)sulfones react efficiently with nucleophiles [28,30,31,36]. Thus, treatment of (*β*-iodovinyl)sulfone **11** with propanethiol (1.0 equiv) in the presence of Et_3_N (1.2 equiv) at rt for 24 h afforded (*β*-thiovinyl)sulfones **20** (60%) as a single *E-*isomer (Scheme 3). Reaction of **11** with diisopropylamine in MeOH (rt, 20 min) gave *β*-(dialkylamino)vinyl sulfone **21** (*E/Z*, ~46:54; 74%).

**Scheme 4:**
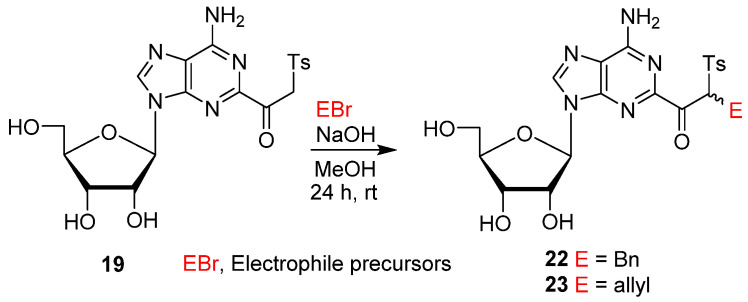
Alkylation of the 2-(*β*-ketosulfone) of adenosine.

Reactivity of *β*-ketosulfone probes was tested in reactions with electrophiles. Thus, treatment of **19** with benzyl bromide (BnBr) in the presence of NaOH (aq) at rt afforded a diastereotopic mixture of the α-monobenzylated product **22** (50/50; 75%) with no α,α-dialkylated byproduct observed (Scheme 4). Alkylation of **19** with allyl bromide gave a diastereotopic mixture of α-allylated ketone **23** (50/50; 68%). These results demonstrate that *β*-ketosulfone and (*β*-iodovinyl)sulfone groups at the C2 position of the adenine ring can act as mechanistically different probes for further modification and potential covalent inhibition of proteins after incorporation into oligonucleotide fragments.

**Scheme 5:**
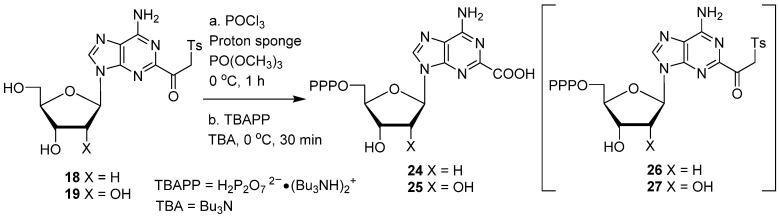
Conversion of 2-(*β*-ketosulfone) group of adenine nucleosides to adenine-2-carboxylate derivatives.

5′-Phosphorylation of **18** with POCl_3_ in the presence of proton sponge followed by treatment with tributylammonium pyrophosphate (TBAPP, 5.0 equiv.) and tributylamine (TBA, 4.0 equiv.) at 0 °C did not provide the expected 5′-triphosphate of *β*-ketosulfone **26** (Scheme 5). Surprisingly, purification of the crude phosphorylation mixture on a Sephadex column with triethylammonium bicarbonate buffer (TEAB) provided the 2-carboxylic acid of 2′-deoxyadenosine-5′-triphosphate **24** (2-CdATP; 25%). Analogous phosphorylation of **19** led to the formation of the 2-carboxylic acid derivative of adenosine-5′-triphosphate **25** (2-CATP; 22%), instead of the expected **27**.

**Scheme 6:**
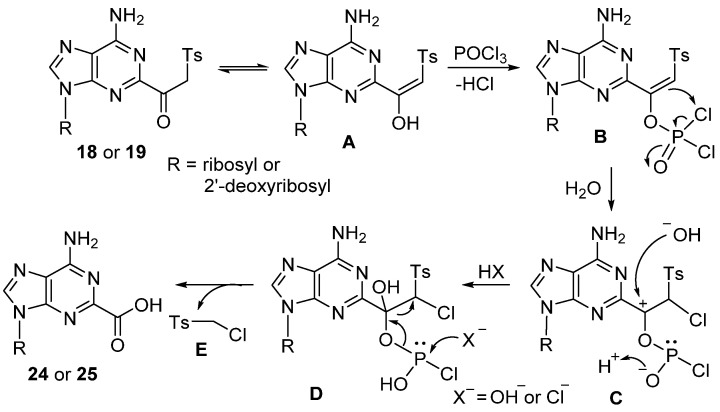
Tentative mechanism for the formation of 2-carboxylic acid of adenine nucleosides from C2 *β*-ketosulfones via electrophilic chlorination. S_N_2 substitution at trivalent phosphorus atom and cleavage of carbon–carbon bond with elimination of (chloromethyl)sulfone fragment.

To confirm the instability of the β-ketosulfone moiety at the adenine C2 position during phosphorylation and/or purification steps, we treated **18** and **19** with the Sephadex resin in 0.5 M TEAB solution at rt overnight. TLC and ^1^H NMR analysis showed stability of **18** and **19** under these conditions, which might indicate that the reagents used for the phosphorylation and/or the presence of the triphosphate group at C5′ might be involved in/facilitate the conversion *β*-ketosulfones to carboxylic acids **24** or **25**. The tentative pathway for this unexpected conversion of the *β*-ketosulfone group to carboxylic acid with the concomitant loss of (chloromethyl)sulfonyl fragment **E** is presented in Scheme 6. Thus, the initial reaction of POCl_3_ with the enol form of *β*-ketosulfones **A** yielded the dichlorophosphate ester **B**. Subsequent intramolecular electrophilic chlorination [46] produced the oxo carbocation **C**. The ensuing S_N_2 substitution at the trivalent phosphorus atom [47] **D** with concomitant elimination of the (chloromethyl)sulfone fragment [48,49] **E** led to the formation of a carboxylic acid. It is noteworthy that adenine nucleosides substituted with *β*-ketosulfone moiety at C8 were stable under the same phosphorylation conditions [31].

**Scheme 7:**
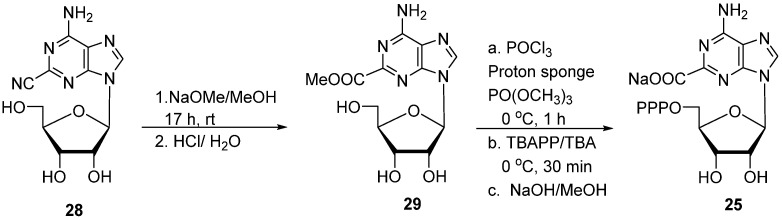
Alternative synthesis of 2-carboxylic acid of adenosine-5′-triphosphate **25**.

To verify the structure of 2-CATP **25**, an independent synthesis of **25** was undertaken. Our initial effort to oxidize 2-methyladenosine [6] with basic KMnO_4_ at reflux failed to produce adenosine-2-carboxylic acid. Also, hydrolysis of 2-cyanoadenosine **28** [50] with NaOH did not succeed in producing adenosine-2-carboxylic acid. However, treatment of **28** with NaOMe, followed by hydrolysis with dilute HCl (aq), afforded the corresponding methyl ester **29** (80%; Scheme 7). 5′-Phosphorylation of **29** and hydrolysis of the carboxylic ester with aqueous NaOH afforded **25** in a 25% yield after Sephadex purification. Triphosphate **25** prepared using both methods (Scheme 5 and Scheme 7) has identical NMR spectra, and its structure was also confirmed by HRMS (High-Resolution Mass Spectrometry). Analogues **24** and **25** can be considered as displaced isoguanosine analogues [51] in which the 2 carboxylate group can be involved in hydrogen bonding, instead of the C2 keto group present in isoguanosine [51,52]. The reactive carboxylic group can be explored for further bioconjugation [53,54,55] with reactive fluorescent amines and DNA–protein crosslink.

**Figure 1:**
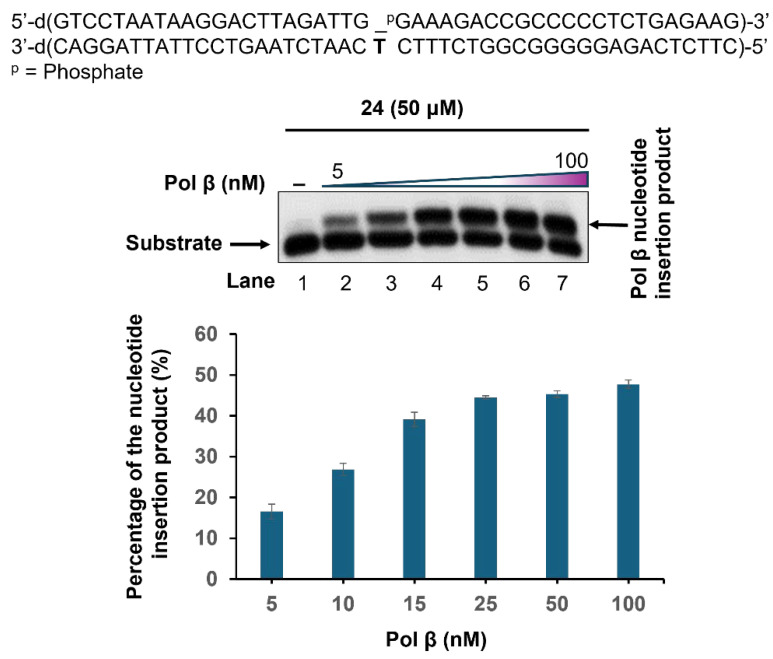
Incorporation of triphosphate **24** by Pol β. The incorporation of **24** at 1 nt gap by Pol β was examined in the presence of 50 nM of 1 nt gap substrate and increasing concentrations of Pol β (5 nM–100 nM). Lane 1 indicates substrate only. Lanes 2–7 show **24** insertion products by Pol β at 5 nM–100 nM. The percentage of **24** insertion products by Pol β was quantified and illustrated in the bar chart below the gel. The substrate was ^32^P-labeled at the 5′-end of the upstream primer. Substrates and products were separated by 15% 7M urea-denaturing polyacrylamide gel and detected by a PhosphorImager. The experiments were repeated at least three times independently. A representative gel image is illustrated.

The incorporation of triphosphate **24** into a duplex DNA by Pol β was examined by incubating a DNA (50 nM) substrate containing a 1 nucleotide (nt) gap and 5′-phosphate with increasing concentrations of Pol β (1 nM–100 nM) in the presence of **24** (50 µM) (Figure 1). The results showed that Pol β readily incorporated **24** to fill in the 1 nt gap at a low concentration of 5 nM (Figure 1, lane 2). Increasing concentrations of Pol β from 5 nM to 25 nM led to significantly elevated incorporation of nucleotide **24** (lanes 2–5). However, Pol β at 50 nM and 100 nM exhibited a similar percentage of nucleotide insertion products as 25 nM (compare lanes 6–7 with lane 5). The results indicate that nucleotide **24** can be readily incorporated into the DNA substrate by a repair DNA polymerase.

It should be noted that the incorporation of **24** by Pol β was less efficient than with analogs containing the β-keto)sulfone group [30,31]. The results may suggest that the carboxylic group in **24** could be more reactive and can react with the amino acid residues in the catalytic center of Pol β and partially inactivate the enzyme, leading to less efficient nucleotide incorporation. This indicates that under cellular physiological conditions, the carboxylate group can be further developed into anticancer and crosslink agents for biomedical applications.

A previous study has shown the incorporation of C2-modified purine nucleotides and the potential application of their fluorescence conjugates in detecting DNA and RNA in cells [22,25]. It is possible that **24** can also be conjugated with a fluorescent tag to be developed as a new probe for DNA staining in cells. Additionally, the modification at C2 can be used to investigate the effect of the functional groups at the position on their insertion by DNA polymerases [22].

## 3. Experimental Section

General Information. ^1^H NMR spectra at 400 MHz and ^13^C NMR at 101 MHz were recorded in DMSO-*d*_6_ or CDCl_3_ unless otherwise noted. All chemical shift values are reported in parts per million (ppm) and referenced to the residual solvent peaks of CDCl_3_ (7.26 ppm), DMSO-*d*_6_ (2.50 ppm), MeOD-*d*_4_ (3.31 ppm) and D_2_O (4.79 ppm) for ^1^H NMR, and referenced to the CDCl_3_ (77.16 ppm), DMSO-*d*_6_ (39.52 ppm) and MeOD-*d*_4_ (49.00 ppm) peaks for the ^13^C NMR spectra, with coupling constant (J) values reported in Hz. HRMS data were recorded in TOF mode, negative or positive, unless otherwise noted. Reaction progress was monitored by TLC on Merck Kieselgel 60-F_254_ sheets, with product detection by 254 nm light. Products were purified by column chromatography using Merck (Boston, MA, USA) Kiselgel 60 (230–400 mesh). Reagent-grade chemicals were used, and solvents were purchased from commercial suppliers and used without further purification unless otherwise specified. DNA oligonucleotides were synthesized by Eurofins Genomics (Louisville, KY, USA). Radionucleotide, ^32^P-ATP (6000 µCi/mmol), was purchased from PerkinElmer Inc. (Boston, MA, USA). Micro Bio-Spin 6 chromatography columns were from Bio-Rad (Hercules, CA, USA). DNA polymerase β (Pol β) was purified as previously described [56]. All other standard chemical reagents were from Sigma–Aldrich (St. Louis, MO, USA) and Thermo Fisher Scientific (Pittsburgh, PA, USA).

*3′,5′-Di-O-(tert-butyldimethylsilyl)-2-ethynyl-2′-deoxyadenosine* (**5**). Step a. Pd(PPh_3_)_2_Cl_2_ (70.0 mg, 0.10 mmol) and Cu(I)I (38.1 mg, 0.2 mmol) were added to dry DMF (50 mL) and Et_3_N (5.0 mL, 3.63 g, 35.9 mmol) in a flame-dried flask equipped with a stirring bar under N_2_ at rt. Then, **3** [34] (3.0 g, 4.95 mmol) was added followed by TMS–acetylene (1.41 mL, 973 mg, 9.90 mmol). The resulting mixture was stirred at 80 °C for 6 h. Volatiles were evaporated, and the residue was purified by column chromatography (20 → 50% EtOAc/hexane) to give TMS-protected alkyne **5** as a brown solid. Step b. The solid was dissolved in anhydrous MeOH (50 mL) and stirred at 0 °C. Anhydrous K_2_CO_3_ (3.2 g, 23.1 mmol) was added portion-wise at room temperature, and the mixture was stirred at rt. After 30 min, the reaction was concentrated under reduced pressure, and the residue was suspended in a mixture of H_2_O (30 mL) and EtOAc (50 mL). The organic layer was washed with brine (50 mL), dried over Na_2_SO_4_, filtered, and concentrated to dryness under vacuum to give a light-yellow crude solid. The solid was recrystallized with hexane to give **5** (2.25 g, 90%, overall) as an off-white solid. ^1^H NMR (400 MHz, CDCl_3_) δ 8.26 (s, 1H), 6.47 (t, *J* = 6.3 Hz, 1H), 6.07 (s, 2H), 4.61 (dt, *J* = 5.9, 3.9 Hz, 1H), 3.99 (q, *J* = 3.5 Hz, 1H), 3.90 (dd, *J* = 11.2, 3.9 Hz, 1H), 3.77 (dd, *J* = 11.2, 3.0 Hz, 1H), 3.00 (s, 1H), 2.58 (dt, *J* = 12.5, 6.1 Hz, 1H), 2.45 (ddd, *J* = 9.1, 6.3, 3.2 Hz, 1H), 0.92 (s, 10H), 0.91 (s, 9H), 0.10 (s, 6H), 0.09 (s, 6H); ^13^C NMR (101 MHz, CDCl_3_) δ 155.49, 149.43, 145.25, 140.19, 119.86, 88.02, 84.54, 82.68, 73.22, 71.84, 62.81, 41.69, 26.09, 25.90, 18.55, 18.14, −4.52, −4.70, −4.98, −5.26, −5.37; HRMS (TOF, ESI) *m/z* calcd for C_24_H_42_N_5_O_3_Si_2_ 504.2821 [M + H]^+^, found 504.2832.

*2′,3′,5′-Tri-O-(tert-butyldimethylsilyl)-2-ethynyladenosine* (**6**). Step a. Treatment of **4** [5] (5.0 g, 6.79 mmol) with TMS–acetylene (1.93 mL, 1.33 g, 13.6 mmol) in the presence of Pd(PPh_3_)_2_Cl_2_ (71.5 mg, 0.102 mmol), Cu(I)I (38.8 mg, 0.204 mmol) and Et_3_N (5.0 mL, 3.63 g, 35.9 mmol) followed by treatment with K_2_CO_3_ (3.2 g, 23.1 mmol; Step b), as described for **5**, gave **6** (3.79 g, 88%, overall) as an off-white solid. ^1^H NMR (400 MHz, CDCl_3_) δ 8.19 (s, 1H), 6.00 (d, *J* = 5.2 Hz, 1H), 4.70 (t, *J* = 4.8 Hz, 1H), 4.31 (t, *J* = 3.8 Hz, 1H), 4.10–4.14 (m, 1H), 4.06 (dd, *J* = 11.2, 4.8 Hz, 1H), 3.78 (dd, *J* = 11.2, 2.8 Hz, 1H), 2.93 (s, 1H), 0.95 (s, 9H), 0.93 (s, 9H), 0.81 (s, 9H), 0.14 (s, 3H), 0.13 (s, 3H), 0.103 (s, 3H), 0.097 (s, 3H), −0.04 (s, 3H), −0.21 (s, 3H); ^13^C NMR (101 MHz, CDCl_3_) δ 155.39, 149.79, 145.27, 140.84, 120.07, 88.84, 85.69, 82.66, 75.79, 72.93, 72.08, 62.60, 26.22, 25.99, 25.84, 18.64, 18.24, 18.02, −4.24, −4.58, −4.62, −4.98, −5.241, −5.238; HRMS (TOF, ESI) *m/z* calcd for C_30_H_56_N_5_O_4_Si_3_ 634.3635 [M + H]^+^, found 634.3641.

*3′,5′-Di-O-(tert-butyldimethylsilyl)-2-(2-iodoethynyl)-2′-deoxyadenosine* (**7**). Sodium acetate (7.4 mg, 0.09 mmol), sodium *p*-toluenesulfinate (32.1 mg, 0.18 mmol), and I_2_ (22.8 mg, 0.09 mmol) were added to a stirring solution of **5** (31 mg, 0.06 mmol) in MeCN at rt. The resulting mixture was then heated at 80 °C for 1.5 h. The reaction mixture was quenched by saturated aqueous Na_2_S_2_O_3_. The volatiles were removed under reduced pressure, and the residue was suspended in a mixture of H_2_O (30 mL) and EtOAc (50 mL). The organic layer was washed with brine (50 mL), dried over Na_2_SO_4_, filtered, and concentrated to dryness under vacuum to give a light-yellow crude solid. The residue was purified by column chromatography (30 → 50% EtOAc/hexane) to give **7** (30 mg, 80%) as an off-white solid. ^1^H NMR (400 MHz, CDCl_3_) δ 8.22 (s, 1H), 6.45 (t, *J* = 6.4 Hz, 1H), 5.92 (s, 2H), 4.61 (dt, *J* = 6.0, 3.6 Hz, 1H), 3.99 (q, *J* = 3.4 Hz, 1H), 3.90 (dd, *J* = 11.2, 4.0 Hz, 1H), 3.77 (dd, *J* = 11.6, 3.2 Hz, 1H), 2.65–2.56 (m, 1H), 2.43 (ddd, *J* = 13.2, 6.0, 4.4 Hz, 1H), 0.92 (s, 9H), 0.91 (s, 9H), 0.10 (s, 12H); ^13^C NMR (101 MHz, CDCl_3_) δ 154.92, 149.31, 145.40, 140.46, 119.76, 93.73, 88.09, 84.69, 71.85, 62.78, 41.62, 26.14, 26.09, 25.96, 25.90, 24.84, 18.58, 18.18, −4.47, −4.54, −4.65, −4.71, −5.20, −5.26, −5.31, −5.37; HRMS (TOF, ESI) *m/z* calcd for C_24_H_41_IN_5_O_3_Si_2_ 630.1787 [M + H]^+^, found 630.1778.

*(E)-3′,5′-Di-O-(tert-butyldimethylsilyl)-2-(1-iodo-2-tosylvinyl)-2′-deoxyadenosine* (**8**). NaOAc (342 mg, 4.17 mmol) and freshly prepared TsI (784 mg, 2.78 mmol) were added to a stirring solution of **5** (700 mg, 1.39 mmol) in THF (15 mL) at rt. After stirring for 40 h, the reaction mixture was quenched with saturated aqueous Na_2_S_2_O_3_. The volatiles were removed under reduced pressure, and the residue was suspended in a mixture of H_2_O (30 mL) and EtOAc (50 mL). The organic layer was washed with brine (50 mL), dried over Na_2_SO_4_, filtered, and concentrated to dryness under vacuum. The residue was purified by column chromatography (30 → 50% EtOAc/hexane) to give **8** (885 mg, 81%) as an off-white solid. ^1^H NMR (400 MHz, CDCl_3_) δ 8.19 (s, 1H), 7.75, (d, *J* = 8.4 Hz, 2H), 7.22 (d, *J* = 7.8 Hz, 2H), 7.23 (s, 1H), 6.41 (t, *J* = 6.4 Hz, 1H), 6.22 (s, 2H), 4.63 (dt, *J* = 6.0, 3.6 Hz, 1H), 4.03 (q, *J* = 3.6 Hz, 1H), 3.92 (dd, *J* = 11.2, 4.4 Hz, 1H), 3.79 (dd, *J* = 10.8, 3.2 Hz, 1H), 2.76–2.68 (m, 1H), 2.44 (ddd, *J* = 13.2, 6.0, 4.0 Hz, 1H), 2.31 (s, 3H), 0.93 (s, 9H), 0.92 (s, 9H), 0.12–0.11 (m, 12H); ^13^C NMR (101 MHz, CDCl_3_) δ 158.29, 155.16, 149.08, 144.94, 140.63, 140.54, 137.22, 129.86, 128.56, 119.33, 110.84, 88.29, 84.89, 72.12, 63.12, 41.46, 26.24, 26.04, 21.18, 18.68, 18.27, −4.36, −4.53, −5.08, −5.17; HRMS (TOF, ESI) *m/z* calcd for C_31_H_49_IN_5_O_5_SSi_2_ 786.2032 [M + H]^+^, found 786.2021.

*(E)-2′,3′,5′-Tri-O-(tert-butyldimethylsilyl)-2-(1-iodo-2-tosylvinyl)adenosine* (**9**). Treatment of **6** (1.5 g, 2.36 mmol) with NaOAc (582 mg, 7.09 mmol) and freshly prepared TsI (1.33 g, 4.72 mmol), as described for **8**, gave **9** (1.99 g, 92%) as an off-white solid. ^1^H NMR (400 MHz, CDCl_3_) δ 8.26 (s, 1H); 7.73 (d, *J* = 8.4 Hz, 2H), 7.23–7.18 (m, 3H), 6.28 (s, 2H), 5.93 (d, *J* = 4.0 Hz, 1H), 4.60 (t, *J* = 4.0 Hz, 1H), 4.34–4.32 (m, 1H), 4.16 (td, *J* = 4.8, 2.8 Hz, 1H), 4.10 (dd, *J* = 11.2, 4.4 Hz, 1H), 3.83 (dd, *J* = 11.2, 2.6 Hz, 1H), 2.30 (s, 3H), 0.97 (s, 9H), 0.93 (s, 9H), 0.84 (s, 9H), 0.17 (s, 3H), 0.16 (s, 3H), 0.11 (s, 3H), 0.09 (s, 3H), 0.02 (s, 3H), −0.07 (s, 3H); ^13^C NMR (101 MHz, CDCl_3_) δ 158.34, 155.04, 149.13, 144.83, 140.68, 140.40, 137.02, 129.74, 128.53, 119.24, 110.06, 89.29, 84.93, 75.83, 71.31, 62.25, 26.29, 26.01, 25.97, 21.70, 18.72, 18.23, 18.07, −4.14, −4.51, −4.60, −4.67, −5.09, −5.17; HRMS (TOF, ESI) *m/z* calcd for C_37_H_63_IN_5_O_6_SSi_3_ 916.2846 [M + H]^+^, found 916.2867.

*(E)-2-(1-Iodo-2-tosylvinyl)-2′-deoxyadenosine* (**10**). CH_3_CO_2_H (1.7 mL, 1.78 g, 29.6 mmol) and NH_4_F (944 mg, 25.5 mmol) were added to a stirring solution of **8** (400 mg, 0.51 mmol) in MeOH (15 mL) at rt. The resulting mixture was stirred at 60 °C for 3.0 h. The volatiles were evaporated at reduced pressure and co-evaporated with acetonitrile (3 × 5 mL) yielding an off-white solid, which was suspended in 20% MeOH in CH_2_Cl_2_. The white precipitate was removed by vacuum filtration, and the mother liquor evaporated at reduced pressure. The residue was purified by column chromatography (5 → 10% MeOH/CH_2_Cl_2_) to give **10** (222 mg, 78%) as a white solid. ^1^H NMR (400 MHz, MeOD-*d*_4_) δ 8.38 (s, 1H), 7.71 (d, *J* = 8.0 Hz, 2H), 7.45 (s, 1H), 7.32 (d, *J* = 8.0 Hz, 2H), 6.42 (t, *J* = 6.8 Hz, 1H), 4.60 (quint, *J* = 2.8 Hz, 1H), 4.09 (q, *J* = 3.2 Hz, 1H), 3.87 (dd, *J* = 12.4, 3.2 Hz, 1H), 3.75 (dd, *J* = 12.0, 3.6 Hz, 1H), 2.86–2.77 (m, 1H), 2.43 (ddd, *J* = 13.6, 6.4, 3.2 Hz, 1H), 2.37 (s, 3H); ^13^C NMR (101 MHz, MeOD-*d*_4_) δ 160.11, 156.90, 149.69, 146.53, 142.38, 141.36, 138.06, 130.82, 129.43, 119.80, 110.85, 89.85, 86.78, 73.04, 63.64, 41.57, 21.64; HRMS (TOF, ESI) *m/z* calcd for C_19_H_21_IN_5_O_5_S 558.0303 [M + H]^+^, found 558.0330.

*(E)-2-(1-Iodo-2-tosylvinyl)adenosine* (**11**). Treatment of **9** (800 mg, 0.87 mmol) with CH_3_CO_2_H (3.1 mL, 3.25 g, 54.2 mmol) and NH_4_F (1.61 g, 43.5 mmol) in MeOH (20 mL), as described for **10**, gave **11** (499 mg, 80%) as a white solid. ^1^H NMR (400 MHz, MeOD-*d*_4_) δ 8.38 (s, 1H), 7.70 (d, *J* = 8.4 Hz, 2H), 7.46 (s, 1H), 7.31 (d, *J* = 8.0 Hz, 2H), 5.98 (d, *J* = 6.4 Hz, 1H), 4.75 (dd, *J* = 6.2, 5.0 Hz, 1H), 4.36 (dd, *J* = 5.0, 3.0 Hz, 1H), 4.20 (q, *J* = 2.8 Hz, 1H), 3.92 (dd, *J* = 12.6, 2.6 Hz, 1H), 3.75 (dd, *J* = 12.6, 3.0 Hz, 1H), 2.35 (s, 3H); ^13^C NMR (101 MHz, MeOD-*d*_4_) δ 160.13, 156.96, 149.72, 146.56, 142.74, 141.48, 137.81, 130.83, 129.49, 119.98, 110.37, 90.97, 87.97, 75.66, 72.59, 63.38, 21.64; HRMS (TOF, ESI) *m/z* calcd for C_19_H_21_IN_5_O_6_S 574.0252 [M + H]^+^, found 574.0253.

*(Z)-2′,3′,5′-Tri-O-(tert-butyldimethylsilyl)-2-(1-amino-2-tosylvinyl)adenosine* (**13**). Iodovinylsulfone **9** (303 mg, 0.33 mmol) was dissolved in methanolic ammonia (1 M, 12 mL), and the resulting mixture was stirred at rt for 2 h. The volatiles were removed under reduced pressure, and the residue was suspended in a mixture of H_2_O (30 mL) and EtOAc (50 mL). The organic layer was washed with brine (50 mL), dried over Na_2_SO_4_, filtered, and evaporated to give **13** (256 mg, 96%) as an off-white solid. ^1^H NMR (400 MHz, CDCl_3_) δ 8.38 (s, 1H), 7.85 (d, *J* = 8.4 Hz, 2H), 7.27 (d, *J* = 7.6 Hz, 2H), 6.9 (s, 2H), 6.27 (s, 1H), 6.04 (d, *J* = 3.6 Hz, 1H), 5.62 (s, 2H), 4.32 (t, *J* = 4.0 Hz, 1H), 4.29 (t, *J* = 4.6 Hz, 1H), 4.13–4.11 (m, 1H), 4.01 (dd, *J* = 11.6, 2.8 Hz, 1H), 3.81 (dd, *J* = 11.6, 2.0 Hz, 1H), 2.39 (s, 3H), 0.96 (s, 9H), 0.92 (s, 9H), 0.79 (s, 9H), 0.16 (s, 3H), 0.14 (s, 3H), 0.09 (s, 3H), 0.07 (s, 3H), −0.05 (s, 3H), −0.18 (s, 3H); ^13^C NMR (101 MHz, CDCl_3_) δ 154.64, 153.41, 149.98, 149.84, 143.04, 141.87, 140.74, 129.62, 126.37, 120.19, 92.90, 88.32, 84.73, 77.02, 71.20, 62.16, 26.27, 25.98, 25.75, 21.64, 18.72, 18.24, 17.96, −4.14, −4.65, −4.67, −4.93, −5.18, −5.23; HRMS (TOF, ESI) *m/z* calcd for C_37_H_65_N_6_O_6_SSi_3_ 805.3989 [M + H]^+^, found 805.3971.

*(Z)-2-(1-Amino-2-tosylvinyl)-2′-deoxyadenosine* (**14**). Iodovinylsulfone **10** (100 mg, 0.18 mmol) was dissolved in methanolic ammonia (1 M, 5 mL), and the resulting mixture was stirred at rt for 2h. The volatiles were evaporated at reduced pressure and co-evaporated with acetonitrile (3 × 5 mL), yielding an off-white solid, which was suspended in 10% MeOH in CH_2_Cl_2_. The off-white precipitate was removed by vacuum filtration, and the mother liquor was evaporated at reduced pressure to give **14** (56 mg, 70%) as a white solid. ^1^H NMR (400 MHz, DMSO-*d*_6_) δ 8.45 (s, 1H), 7.80 (d, *J* = 8.3 Hz, 2H), 7.55 (s, 2H), 7.39 (d, *J* = 7.6 Hz, 2H), 6.98 (s, 2H), 6.40 (t, *J* = 6.8 Hz, 1H), 6.00 (s, 1H), 5.34 (s, 1H), 4.93 (s, 1H), 4.40 (s, 1H), 3.87–2.82 (m, 1H), 3.59–3.49 (m, 2H), 2.70–2.63 (m, 1H), 2.37 (s, 3H), 2.30 (ddd, *J* = 13.6, 6.4, 3.6 Hz, 1H); ^13^C NMR (101 MHz, MeOD-d_4_) δ 156.75, 154.60, 152.29, 150.71, 144.65, 143.07, 142.23, 130.66, 127.00, 120.50, 91.72, 89.22, 85.62, 72.47, 63.12, 41.34, 21.46; HRMS (TOF, ESI) *m/z* calcd for C_19_H_23_N_6_O_5_S 447.1445 [M + H]^+^, found 447.1445.

*(Z)-2-(1-Amino-2-tosylvinyl)adenosine* (**15**). Treatment of **11** (150 mg, 0.26 mmol) with methanolic ammonia (1 M, 10 mL), as described for **14**, gave **15** (85 mg, 70%) as a white solid. ^1^H NMR (400 MHz, DMSO-*d*_6_) δ 8.47 (s, 1H), 7.86–7.74 (m, 2H), 7.57 (s, 2H), 7.43–7.34 (m, 2H), 7.06 (s, 1H), 6.98 (s, 2H), 6.00 (s, 1H), 5.94 (d, *J* = 5.8 Hz, 1H), 5.48 (d, *J* = 6.2 Hz, 1H), 5.21 (d, *J* = 5.0 Hz, 1H), 5.02 (t, *J* = 5.5 Hz, 1H), 4.52 (q, *J* = 5.8 Hz, 1H), 4.14 (td, *J* = 5.0, 3.5 Hz, 1H), 3.93 (q, *J* = 3.9 Hz, 1H), 3.64 (dt, *J* = 11.9, 4.9 Hz, 1H), 3.55 (dt, *J* = 11.9, 4.8 Hz, 1H), 2.37 (s, 3H); ^13^C NMR (101 MHz, CDCl_3_) δ ^13^C NMR (101 MHz, DMSO-*d*_6_) δ 155.39, 152.37, 150.23, 149.45, 142.81, 141.78, 140.90, 129.70, 125.63, 119.32, 90.73, 87.05, 85.52, 73.95, 70.28, 61.29, 54.93, 20.99; HRMS (TOF, ESI) *m/z* calcd for C_19_H_23_N_6_O_6_S 463.1394 [M + H]^+^, found 463.1383.

*3′,5′-Di-O-(tert-butyldimethylsilyl)-2-(2-tosylacetyl)-2′-deoxyadenosine* (**16**). Step a. Treatment of iodovinylsulfone **8** (200 mg, 0.25 mmol) with methanolic ammonia (1 M, 10 mL), as described for **13**, gave (*Z*)-3′,5′-Di-*O*-(*tert*-butyldimethylsilyl)-2-(1-amino-2-tosylvinyl)-2′-deoxyadenosine **12** (160 mg, 95%) as an off-white solid of sufficient purity to be used directly in the next step. Step b. CH_3_CO_2_H/H_2_O (1:1, 0.5 mL) was added to a stirring solution of **12** (150 mg, 0.22 mmol) in MeOH (5 mL) at rt. The resulting solution was stirred at rt for 8.0 h. The volatiles were removed under reduced pressure, and the residue was suspended in mixture of H_2_O (20 mL) and EtOAc (30 mL). The organic layer was washed with brine (20 mL), dried over Na_2_SO_4_, filtered, and concentrated to dryness under vacuum. The residue was purified by column chromatography (40 → 50% EtOAc/hexane) to give **16** (129 mg, 86%) as a white solid. ^1^H NMR (400 MHz, CDCl_3_) δ 8.34 (s, 1H), 7.77 (d, *J* = 8.4 Hz, 2H), 7.26 (d, *J* = 8.0 Hz, 2H), 6.69 (s, 2H),6.42 (t, *J* = 6.2 Hz, 1H), 5.10 (s, 2H), 4.62–4.57 (m, 1H), 4.03 (q, *J* = 3.2 Hz, 1H), 3.89 (dd, *J* = 11.2, 3.6 Hz, 1H), 3.80 (dd, *J* = 11.2, 2.8 Hz, 1H), 2.54–2.46 (m, 2H), 2.36 (s, 3H), 0.93 (s, 9H), 0.92 (s, 9H), 0.12 (s, 3H), 0.12 (s, 3H), 0.11 (s, 6H); ^13^C NMR (101 MHz, CDCl_3_) δ 187.75, 155.82, 153.54, 148.75, 145.09, 141.63, 136.42, 129.75, 128.77, 121.08, 88.12, 84.44, 71.70, 62.80, 62.69, 42.16, 26.09, 25.89, 21.72, 18.55, 18.14, −4.46, −4.66, −5.25, −5.35; HRMS (TOF, ESI) *m/z* calcd for C_31_H_50_N_5_O_6_SSi_2_ 676.3015 [M + H]^+^, found 676.3001.

*2′,3′,5′-Tri-O-(tert-butyldimethylsilyl)-2-(2-tosylacetyl)adenosine* (**17**). Treatment of **13** (250 mg, 0.31 mmol) with CH_3_CO_2_H/H_2_O (1:1, 1.0 mL), as described for **16** (Step b), gave **17** (213 mg, 85%) as a white solid. ^1^H NMR (400 MHz, CDCl_3_) δ 8.46 (s, 1H), 7.76 (d, *J* = 8.4 Hz, 2H), 7.25 (d, *J* = 8.0 Hz, 2H), 6.78 (s,2H), 5.98 (d, *J* = 4.0 Hz, 1H), 5.38 (d, *J* = 14.0 Hz, 1H), 4.76 (d, *J* = 14.0 Hz, 1H), 4.37 (t, *J* = 4.4 Hz, 1H), 4.32 (t, *J* = 4.4 Hz, 1H), 4.18–4.15 (m, 1H), 4.05 (dd, *J* = 11.6, 3.0 Hz, 1H), 3.84 (dd, *J* = 11.6, 2.4 Hz, 1H), 2.35 (s, 3H), 0.98 (s, 9H), 0.94 (s, 9H), 0.81 (s, 9H), 0.18 (s, 3H), 0.16 (s, 3H), 0.12 (s, 3H), 0.10 (s, 3H), −0.10 (s, 3H), −0.19 (s, 3H); ^13^C NMR (101 MHz, CDCl_3_) δ 187.62, 155.98, 153.54, 148.95, 145.14, 141.93, 136.40, 129.75, 128.78, 121.22, 88.25, 85.18, 77.06, 71.54, 62.64, 62.37, 26.26, 25.98, 25.75, 21.70, 18.70, 18.24, 17.96, −4.15, −4.58, −4.65, −4.72, −5.19, −5.23; HRMS (TOF, ESI) *m/z* calcd for C_37_H_64_N_5_O_7_SSi_3_ 806.3829 [M + H]^+^, found 806.3833.

*2-(2-Tosylacetyl)-2′-deoxyadenosine* (**18**). Method A. TBAF [330 µL, 0.33 mmol (1.0 M in THF)] was added to a stirring solution of **16** (101 mg, 0.15 mmol) in THF (5 mL) at 0 °C, and the resulting mixture was stirred at rt for 1 h. The volatiles were evaporated, and the residue was column-chromatographed (5 → 10% MeOH/CH_2_Cl_2_) to give **18** (58 mg, 87%) as a white solid. ^1^H NMR (400 MHz, DMSO-*d*_6_) δ 8.56 (s, 1H), 7.73 (d, *J* = 8.4 Hz, 2H), 7.68 (s, 2H), 7.38 (d, *J* = 8.0 Hz, 2H), 6.33 (t, *J* = 6.8 Hz, 1H), 5.37 (d, *J* = 4.0 Hz, 1H), 5.30 (d, *J* = 2.4 Hz, 2H), 4.94 (t, *J* = 5.6 Hz, 1H), 4.44–3.38 (m, 1H), 3.88 (q, *J* = 4.4 Hz, 1H), 3.63–3.56 (m 1H), 3.55–3.49 (m 1H), 2.73–2.66 (m, 1H), 2.35 (s, 3H), 2.32–2.27 (m, 1H); ^13^C NMR (101 MHz, MeOD-d_4_) δ 189.15, 157.05, 154.60, 150.04, 146.60, 143.82, 137.78, 130.72, 129.58, 121.66, 89.53, 86.28, 72.62, 63.26, 54.81, 41.64, 21.50; HRMS (TOF, ESI) *m/z* calcd for C_19_H_22_N_5_O_6_S 448.1285 [M + H]^+^, found 448.1284.

Method B. Treatment of **14** (67 mg, 0.15 mmol) with CH_3_CO_2_H/H_2_O (1:1, 0.5 mL), as described for **16** (Step b), gave **18** (56 mg, 84%).

*2-(2-Tosylacetyl)adenosine* (**19**). Method A. Treatment of **17** (202 mg, 0.25 mmol) in THF (10 mL) with TBAF [830 µL, 0.83 mmol (1.0 M in THF)], as described for **18**, gave **19** (102 mg, 88%) as a white solid. ^1^H NMR (400 MHz, MeOD-*d*_4_) δ 8.52 (s, 1H), 7.72 (d, *J* = 8.0 Hz, 2H), 7.29 (d, *J* = 8.0 Hz, 2H), 6.00 (d, *J* = 5.6 Hz, 1H), 4.63 (t, *J* = 5.6 Hz, 1H), 4.35 (t, *J* = 4.4 Hz, 1H), 4.16 (q, *J* = 3.2 Hz, 1H), 3.91 (dd, *J* = 12.4, 2.8 Hz, 1H), 3.79 (dd, *J* = 12.6, 3.4 Hz, 1H), 2.31 (s, 3H); ^13^C NMR (101 MHz, MeOD-*d*_4_) δ 189.07, 157.11, 154.66, 150.28, 146.65, 143.96, 137.70, 130.73, 129.62, 121.79, 90.59, 87.38, 76.07, 71.93, 62.86, 21.49; HRMS (TOF, ESI) *m/z* calcd for C_19_H_22_N_5_O_7_S 464.1234 [M + H]^+^, found 464.1233.

Method B. Treatment of **15** (69.5 mg, 0.15 mmol) with CH_3_CO_2_H/H_2_O (1:1, 1.0 mL), as described for **16** (Step b), gave **19** (57 mg, 82%).

*(E)-2-(1-Propanethio-2-tosylvinyl)adenosine* (**20**). Et_3_N (9.0 μL, 6.6 mg, 0.065 mmol) and PrSH (4.8 μL, 4.1 mg, 0.054 mmol) were sequentially added to a stirred solution of **11** (31 mg, 0.054 mmol) in MeOH (2 mL) at ambient temperature. After 24 h, the volatiles were evaporated, and the residue was purified by silica gel column chromatography [MeOH/DCM (dichloromethane); 2 → 5%] to give **20** (17 mg, 60%) as a white solid. ^1^H NMR (400 MHz, MeOD-*d*_4_) δ 8.41 (s, 1H), 8.05 (s, 1H), 7.89 (d, *J* = 8.4 Hz, 2H), 7.45 (d, *J* = 7.7 Hz, 2H), 6.01 (d, *J* = 5.9 Hz, 1H), 4.73 (t, *J* = 5.5 Hz, 1H), 4.33 (dd, *J* = 5.1, 3.4 Hz, 1H), 4.13 (q, *J* = 3.2 Hz, 1H), 3.86 (dd, *J* = 12.4, 2.9 Hz, 1H), 3.77–3.73 (m, 1H), 2.73–2.68 (m, 2H), 2.46 (s, 3H), 1.26–1.20 (m, 2H), 0.67 (t, *J* = 7.3 Hz, 3H). ^13^C NMR (101 MHz, MeOD-*d*_4_) δ 157.14, 157.02, 150.64, 146.44, 145.63, 142.94, 141.93, 136.90, 130.76, 130.26, 119.80, 90.81, 87.65, 75.62, 72.33, 63.21, 39.16, 23.17, 21.59, 13.32; HRMS (TOF, ESI) *m/z* calcd for C_22_H_28_N_5_O_6_S_2_ 522.1476 [M + H]^+^, found 522.1477.

*(E/Z)-2-(1-Diisopropylamino-2-tosylvinyl)adenosine* (**21**). (Me_2_CH)_2_NH (17 μL, 12.3 mg, 0.12 mmol) was added to a stirred solution of **11** (30 mg, 0.052 mmol) in MeOH (3 mL) at ambient temperature. After 30 min, the volatiles were evaporated, and the residue was purified by silica gel column chromatography (MeOH/DCM; 2 → 5%) to give **21** (*E/Z*, ~46:54; 21 mg, 74%) as a white solid. ^1^H NMR (400 MHz, DMSO-*d*_6_) δ 8.42 (s, 0.46H), 8.41 (s, 0.54H), 7.59 (d, *J* = 8.4 Hz, 2H), 7.50 (s, 0.92H), 7.48 (s, 1.08H) 7.35 (d, *J* = 8.0 Hz, 0.92H), 7.28 (d, *J* = 8.0 Hz, 1.08H), 5.89 (d, *J* = 6.4 Hz, 0.46H), 5.83 (d, *J* = 6.4 Hz, 0.54H), 5.53 (dd, *J* = 6.0, 2.8 Hz, 0.46H), 5.40 (d, *J* = 6.4 Hz, 0.54H) 5.25 (dt, *J* = 7.9, 3.5 Hz, 2H), 4.97 (s, 0.54H), 4.94 (s, 0.46H), 4.59–4.51 (m, 1H), 4.14 (qd, *J* = 5.0, 3.3, 2.2 Hz, 1H), 4.02 (q, *J* = 3.2 Hz, 0.54H), 3.95 (q, *J* = 3.2 Hz, 0.46H) 3.71–3.47 (m, 3.46H), 3.25 (d, *J* = 6.4 Hz, 0.54H), 2.36 (s, 3H), 1.16 (br s, 12H). ^13^C NMR (101 MHz, DMSO) δ 155.81, 155.72, 154.11, 153.76, 149.31, 149.22, 143.70, 143.63, 141.01, 140.99, 139.96, 139.87, 129.30, 129.00, 126.05, 118.46, 118.45, 93.58, 93.40, 87.42, 87.09, 86.11, 85.97, 73.96, 73.80, 70.90, 70.76, 61.72, 53.21, 21.01, 20.96, 20.20, 19.58; HRMS (TOF, ESI) *m/z* calcd for C_25_H_35_N_6_O_6_S 547.2333 [M + H]^+^, found 547.2330.

*2-(2-Benzyl-2-tosylacetyl)adenosine* (**22**). Aqueous NaOH solution (1 M, 130 µL, 0.13 mmol) was added to a stirred solution of **19** (30 mg, 0.065 mmol) in MeOH (2 mL) at rt. After 30 min, BnBr (15.4 µL, 22.3 mg, 0.13 mmol) was added, and the resulting mixture was stirred for 24 h. The reaction mixture was then neutralized with dil. HCl to pH ~7, and the volatiles were evaporated. The residue was column-chromatographed (5 → 10% MeOH/DCM) to give **22** (27 mg, 75%) as a 50:50 mixture of diastereomers. ^1^H NMR (400 MHz, MeOD-*d*_4_) δ 8.48 (s, 0.5H), 8.46 (s, 0.5H), 7.67 (d, *J* = 8.0 Hz, 1H), 7.64 (d, *J* = 8.0 Hz, 1H), 7.29–7.06 (m, 7.5H), 6.52–6.45 (m, 0.5H), 5.98 (t, *J* = 5.2 Hz, 1H), 4.61 (t, *J* = 5.2 Hz, 1H), 4.36–4.32 (m, 1H), 4.19 (“q”, *J* = 3.2 Hz, 0.5H), 4.16 (“q”, *J* = 3.2 Hz, 0.5H), 3.94–3.89 (m, 1H), 3.80–3.76 (m, 1H), 3.52–3.42 (m, 2H), 2.25 (s, 1.5H), 2.22 (s, 1.5H); ^13^C NMR (101 MHz, MeOD-*d*_4_) δ 192.47, 192.26, 156.89, 155.13, 154.99, 150.12, 150.06, 146.92, 144.05, 144.02, 137.65, 137.62, 137.60, 137.57, 136.16, 136.09, 130.69, 130.48, 130.36, 130.05, 130.03, 129.63, 127.93, 127.90, 121.66, 90.71, 90.65, 87.50, 87.46, 75.98, 75.90, 72.11, 72.02, 70.39, 63.07, 62.97, 32.72, 32.44, 21.46, 21.44; HRMS (TOF, ESI) *m/z* calcd for C_26_H_28_N_5_O_7_S 554.1704 [M + H]^+^, found 554.1704.

*2-(2-Allyl-2-tosylacetyl)adenosine* (**23**). Aqueous NaOH solution (1 M, 130 µL, 0.13 mmol) was added to a stirred solution of **19** (30 mg, 0.065 mmol) in MeOH (2 mL) at rt. After 30 min, allyl bromide (11.2 µL 15.7 mg, 0.13 mmol) was added, and the resulting mixture was stirred for 24 h. The reaction mixture was then neutralized with dil. HCl to pH~7, and the volatiles were evaporated. The residue was column-chromatographed (5 → 10% MeOH/DCM) to give **23** (22.3 mg, 68%) as a 50:50 mixture of diastereomers in the form of a white solid. ^1^H NMR (400 MHz, MeOD-*d*_4_) δ ^1^H NMR (400 MHz, MeOD-*d*_4_) δ 8.52 (s, 0.5H), 8.51 (s, 0.5H), 7.69–7.58 (m, 2H), 7.28–7.17 (m, 2H), 6.23 (ddd, *J* = 14.4, 10.4, 4.4 Hz, 1H), 6.04 (d, *J* = 5.6 Hz, 0.5H), 6.00 (d, *J* = 5.6 Hz, 0.5H), 5.82–5.70 (m, 1H), 5.12 (q, *J* = 1.6 Hz, 0.5H), 5.10 (q, *J* = 1.6 Hz, 0.5H), 5.09 (t, *J* = 1.2 Hz, 1H), 4.98 (t, *J* = 1.2 Hz, 1H), 4.65 (dt, *J* = 6.4, 5.2 Hz, 1H), 4.35 (ddd, *J* = 5.2, 3.6, 1.6 Hz, 1H), 4.19 (“q”, *J* = 3.2 Hz, 0.5H), 4.17 (“q”, *J* = 3.2 Hz, 0.5H), 3.92 (“ddd”, *J* = 12.4, 7.6, 2.8 Hz, 1H), 3.82–3.77 (m, 1H), 2.99–2.85 (m, 2H), 2.25 (s, 1.5H), 2.22 (s, 1.5H); ^13^C NMR (101 MHz, MeOD-*d*_4_) δ 192.40, 192.22, 157.04, 155.29, 155.14, 150.30, 150.19, 146.91, 144.07, 143.97, 136.16, 136.08, 133.98, 133.95, 133.94, 133.91, 130.65, 130.46, 130.33, 121.72, 118.65, 118.63, 90.64, 90.59, 87.55, 87.52, 76.07, 76.03, 72.11, 72.06, 68.70, 68.61, 63.03, 62.95, 31.14, 31.05, 30.95, 30.86, 21.44, 21.43; HRMS (TOF, ESI) *m/z* calcd for C_22_H_26_N_5_O_7_S 504.1547 [M + H]^+^, found 504.1545.

*2′-Deoxyadenosine-2-carboxylic acid 5′-O-triphosphate* (**24**). (MeO)_3_PO (1.0 mL; dried over 3A molecular sieves) was added to the flame-dried flask containing 8-(*β*-keto)sulfone **18** [30 mg, 0.067 mmol; dried in vacuum (40 °C, over P_2_O_5_)] and proton sponge (21.5 mg, 0.10 mmol), and the resulting solution was stirred at 0 °C for 5 min under an Ar atmosphere. Freshly distilled POCl_3_ (8.1 μL, 13.4 mg, 0.087 mmol) was then added, and stirring was continued for 1.0 h at 0 °C. The mixture of tributylammonium pyrophosphate (TBAPP; 0.5 M/dimethylformamide (DMF); 670 μL, 0.34 mmol) and Bu_3_N (49.7 mg, 0.27 mmol) was added and stirred for another 30 min at 0 °C. The reaction mixture was quenched by adjusting pH to 7.5–7.8 with triethylammonium bicarbonate (TEAB) buffer (2 M, several drops). The residue was dissolved in water (5 mL) and was extracted with EtOAc (3 × 5 mL). The water layer was evaporated and co-evaporated (three times) with a mixture of EtOH/H_2_O (1:1, 5 mL). The residue was chromatographed on a DEAE-Sephadex A-25 column with TEAB (0.1 → 0.6 M), and the appropriate fractions (TLC, R*_f_* 0.28; *i*-PrOH/H_2_O/NH_4_OH, 5:2:3) were evaporated in vacuum and co-evaporated three times with a mixture of EtOH/H_2_O (1:1, 10 mL) to remove excess of TEAB salt to give **24** as a triethylammonium salt, which was then converted to sodium salt **24** (11.0 mg, 25%) with Dowex-Na^+^. ^1^H NMR (400 MHz, D_2_O) *δ* 8.56 (s, 1H), 6.61 (s, 1H), 4.89 (s, 1H), 4.32–4.13 (m, 3H), 2.85–2.74 (m, 1H), 2.69–2.58 (m, 1H); ^31^P NMR (162 MHz, D2O) *δ* −10.89 (d, *J* = 19.8 Hz), −11.44 (d, *J* = 20.1 Hz), −23.24 (t, *J* = 19.8 Hz); HRMS (TOF, ESI) *m/z* calcd for C_11_H_15_N_5_O_14_P_3_ 533.9834 [M − H]^−^, found 533.9830.

*Adenosine-2-carboxylic acid 5′-O-triphosphate* (**25**). Method A: Treatment of **19** (31 mg, 0.067 mmol) with (MeO)_3_PO (1.0 mL), proton sponge (21.5 mg, 0.10 mmol), and freshly distilled POCl_3_ (8.1 μL, 13.4 mg, 0.087 mmol), followed by a mixture of tributylammonium pyrophosphate (TBAPP; 0.5 M/dimethylformamide (DMF); 670 μL, 0.34 mmol) and Bu_3_N (49.7 mg, 0.27 mmol), as described for **24**, gave **25** (9.6 mg, 22%) as a triethylammonium salt.

Method B. (MeO)_3_PO (1.0 mL; dried over 3A molecular sieves) was added to the flame-dried flask containing **29** [40 mg, 0.12 mmol; dried in vacuum (40 °C, over P_2_O_5_)] and proton sponge (38.6 mg, 0.18 mmol), and the resulting solution was stirred at 0 °C for 5 min under an Ar atmosphere. Freshly distilled POCl_3_ (14.6 μL, 24.1 mg, 0.16 mmol) was then added, and stirring was continued for 1.0 h at 0 °C. The mixture of TBAPP (0.5 M/DMF; 1.2 mL, 0.6 mmol) and Bu_3_N (115 μL, 89.5 mg, 0.48 mmol) was added and stirred for another 30 min at 0 °C. The reaction mixture was quenched by adjusting pH to 7.5–7.8 with TEAB buffer (2 M, several drops). The residue was dissolved in water (5 mL) and was extracted with EtOAc (3 × 5 mL). The water layer was evaporated and co-evaporated (three times) with a mixture of EtOH/H_2_O (1:1, 5 mL). The residue was dissolved in MeOH (2 mL). Aqueous NaOH (1 M, 120 μL) was added, and the reaction mixture was stirred rt for 3 h. The reaction mixture was quenched by adjusting pH to 7.0 with HCl (1 M). The residue was chromatographed on a DEAE-Sephadex A-25 column with TEAB (0.1 → 0.6 M), and the appropriate fractions (TLC, R*_f_* 0.31; *i*-PrOH/H_2_O/NH_4_OH, 5:2:3) were evaporated in vacuum and co-evaporated three times with a mixture of EtOH/H_2_O (1:1, 10 mL) to remove excess of TEAB salt to give **25** as a triethylammonium salt. ^1^H NMR (400 MHz, D_2_O) *δ* 8.63 (s, 1H), 6.23 (s, 1H), 4.75 (s, 1H), 4.62 (d, *J* = 4.7 Hz, 1H), 4.40 (s, 1H), 4.29 (dd, *J* = 19.6, 13.3 Hz, 2H); ^31^P NMR (162 MHz, D_2_O) *δ* −5.57 (d, *J* = 19.4 Hz), −10.90 (d, *J* = 19.1 Hz), −21.34 (t, *J* = 19.2 Hz); HRMS (TOF, ESI) *m/z* calcd for C_11_H_15_N_5_O_15_P_3_ 549.9783 [M − H]^−^, found 549.9792.

*2-(Methoxycarbonyl)adenosine* (**29**). A mixture of **28** [50] (100 mg, 0.34 mmol) and NaOMe (5.4 M; 95 μL, 0.51 mmol) in MeOH (10 mL) was stirred at rt for 15 h. After neutralization with Dowex 50 (H^+^) and filtration of resin, the volatiles were removed at reduced pressure. The residue was dissolved in a mixture of MeOH/H_2_O (10 mL; 1:1) and 1.0 M HCl (340 μL, 0.34 mmol) was added. The mixture was stirred at rt for 2 h. After neutralization with 1.0 M NaOH, the volatiles were removed at reduced pressure, and the residue was column-chromatographed (10 → 20% MeOH/DCM) to give **29** (89 mg, 80%) as a white solid. ^1^H NMR (400 MHz, DMSO-*d*_6_) δ 8.53 (s, 1H), 7.71 (s, 2H), 5.93 (d, *J* = 6.4 Hz, 1H), 5.47 (d, *J* = 6.0 Hz, 1H), 5.22 (d, *J* = 4.7 Hz, 1H), 5.05 (dd, *J* = 6.4, 5.2 Hz, 1H), 4.62–4.56 (m, 1H), 4.15 (td, *J* = 4.8, 2.8 Hz, 1H), 3.96 (q, *J* = 3.6 Hz, 1H), 3.84 (s, 3H), 3.71–3.64 (m, 1H), 3.56 (ddd, *J* = 12.0, 6.4, 4.0 Hz, 1H); ^13^C NMR (101 MHz, DMSO-*d*_6_) *δ* 164.47, 156.15, 150.61, 149.47, 141.85, 120.12, 87.41, 86.08, 73.84, 70.73, 61.67, 52.57. HRMS (TOF, ESI) *m/z* calcd for C_12_H_16_N_5_O_6_ 326.1095 [M + H]^+^, found 326.1012.

*Incorporation of the nucleotide analog 24 by Pol β*

The incorporation of **24** by Pol β was examined with the substrate containing a 1 nt-gap with a 5′-phosphate at the downstream primer. The substrate was constructed by annealing the 5′-end 32P-labeled upstream primer and downstream primer with a template at the ratio of 1:2:2. The upstream primer was 5′-end labeled with ^32^P using T4 polynucleotide kinase according to the protocol provided by New England Biolabs (Ipswich, MA, USA). Pol β incorporation of 24 was measured by incubating a 50 nM substrate with increasing concentrations of Pol β (5–100 nM) in the presence of 50 μM of the nucleotide analog at 37 °C for 30 min. The reaction mixture was assembled in a 10 µL reaction buffer containing 50 mM Tris-HCl, pH 7.5, 50 mM KCl, 0.1 mM EDTA, 0.1 mg/mL bovine serum albumin, and 0.01% Nonidet P-40 in the presence of 5 mM MgCl_2_. The enzyme reaction was stopped using 2x stopping buffer containing 95% formamide and 10 mM EDTA, 0.05% (*w*/*v*) bromophenol blue and 0.05% (*w*/*v*) xylene cyanol, followed by incubation at 95 °C for 5 min. Substrates and products were separated by a 15% 7M urea-denaturing polyacrylamide gel and detected by a PhosphorImager. All experiments were repeated at least three times independently.

## 4. Conclusions

We have developed two probes attached to the C2 position of adenosine and 2′-deoxyadenosine that are mechanistically different. The 2-(*β*-iodovinyl)sulfone probe efficiently reacts with nucleophiles, such as amines or thiols, via a conjugated addition–elimination pathway and can be efficiently converted to 2-(*β*-keto)sulfones. The 2-(*β*-keto)sulfone probe instead reacts with electrophiles such as alkyl halides. The aryl ring in sulfone group can be modified to allow for further bioconjugation and/or crosslinking. Adenine nucleosides with a *β*-ketosulfone group at the C2 position, during conversion to their 5′-triphosphate, undergo an unexpected conversion to 2-carboxylic acid nucleotides. The 2-carboxylic acid of 2′-deoxyadenosine-5′-triphosphate is efficiently incorporated by human DNA polymerase into a double-stranded DNA oligomer containing a one-nucleotide gap.

## Figures and Tables

**Table 1:** Synthesis of C2-modified *β*-iodovinylsulfones of 2′-deoxyadenosine and adenosine.

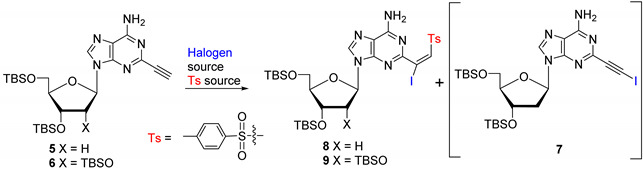							
Entry	Halogen Source	Ts Source	Solvent	Additive	Temp, Time	Product	Yields (%)
1	I_2_	TsNa	MeCN	NaOAc	80 °C, 1.5 h	**7**	80
2	I_2_	TsNa	H_2_O		80 °C, 1.5 h	**7**	40
3	I_2_	TsNa	H_2_O/MeCN		80 °C, 1.5 h	**7**	60
4	KI	TsNHNH_2_	DMSO	Bz_2_O	rt, 15 h	**8**	0 ^[a]^
5	CuI	TsNHNH_2_	DMSO	Bz_2_O	rt, 15 h	**8**	0
6	NaI	TsNa	MeCN	CAN ^[b]^	80 °C, 2 h	**8**	0
7	KI	TsNa	MeCN	PhI(OAc)_2_	80 °C, 2 h	**8**	0
8		TsI	THF		rt, 40 h	**8**	0 ^[c]^
9		TsI	THF	NaOAc	rt, 40 h	**8**	80
10		TsI	THF	NaOAc	rt, 40 h	**9**	9

^[a]^ Partial deprotection occurs. ^[b]^ Ceric ammonium nitrate (CAN). ^[c]^ Glycosylic bond cleavage was observed.

## Data Availability

Data are contained within the article and Supplementary Materials.

## Supplementary Materials

The following supporting information can be downloaded at https://www.mdpi.com/article/10.3390/molecules30061358/s1; copies of ^1^H, ^13^C, and ^31^P NMR spectra.
