# Supplementary material for: Synthesis of Adenine Nucleosides with a Reactive (β-Iodovinyl)sulfone or (β-Keto)sulfone Group at the C2 Position and Their Polymerase-Catalyzed Incorporation into DNA
Source: Molecules. 2025 Mar 18;30(6):1358. doi: 10.3390/molecules30061358 (PMC11944392; doi:10.3390/molecules30061358)
Supplement: Supplementary file 1 [file molecules-30-01358-s001.zip › molecules-3506290-supplementary.pdf]

Adenine Nucleosides with a Reactive (*β*-Iodovinyl)sulfone or (*β*-Keto)sulfone Group at the C2 Positions and Polymerase-Catalyzed Incorporation into DNA

A. Hasan Howlader,[1] Richard Fernandez[1], Pawlos S. Tsegay[1], Yuan Liu[1,2] and Stanislaw F. Wnuk[1,*]

[1]Department of Chemistry and Biochemistry and [2]Biomolecular Sciences Institute, Florida International University, Miami, Florida 33199, United States

SUPPORTIVE INFORMATION

^{1}H, ^{13}C, and ^{31}P NMR spectra of the compounds S2-S22

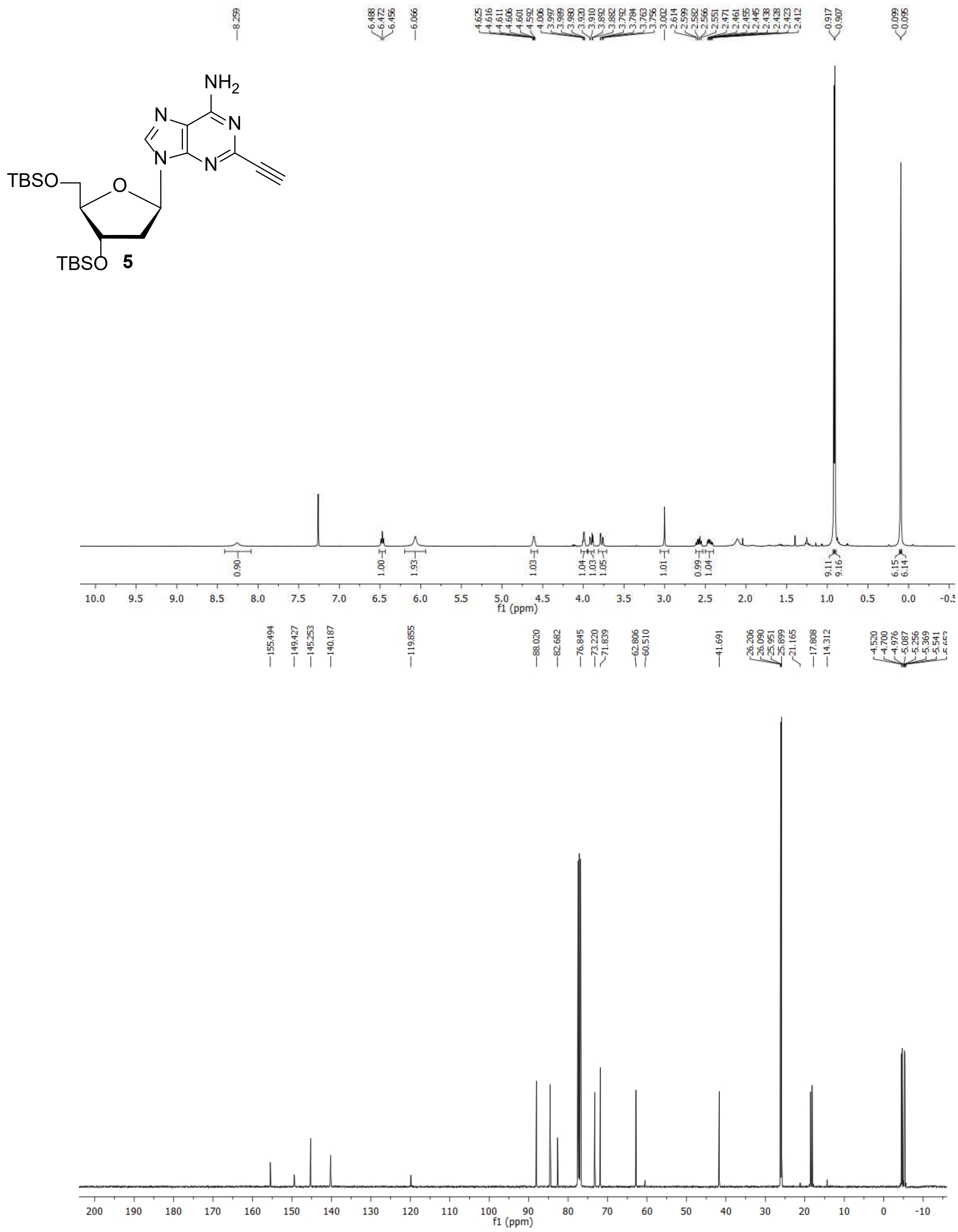

Figure S1. ^{1}H NMR and ^{13}C NMR of compound **5** in $CDCl_3$.

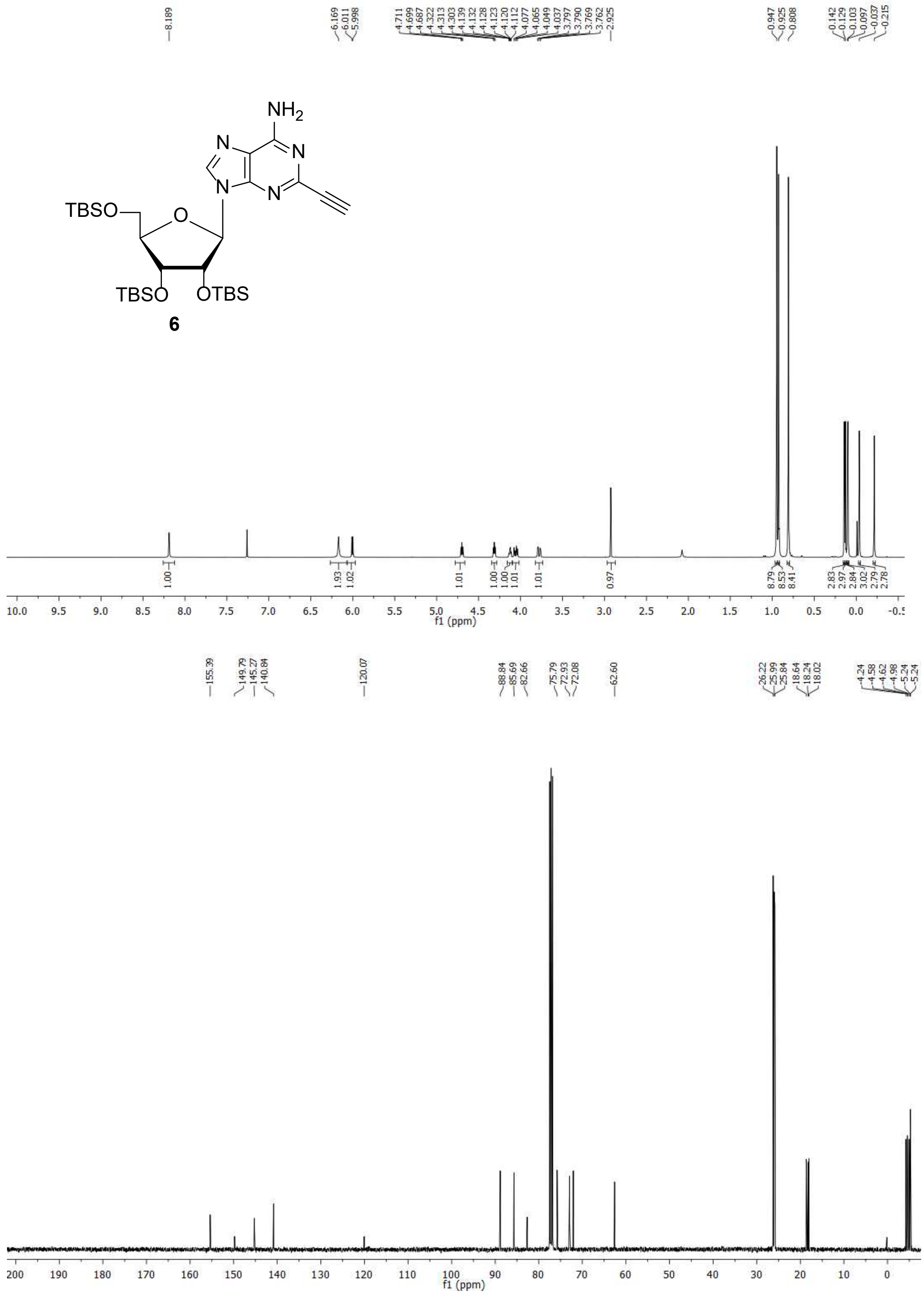

Figure S2. ^{1}H NMR and ^{13}C NMR of compound **6** in $CDCl_3$.

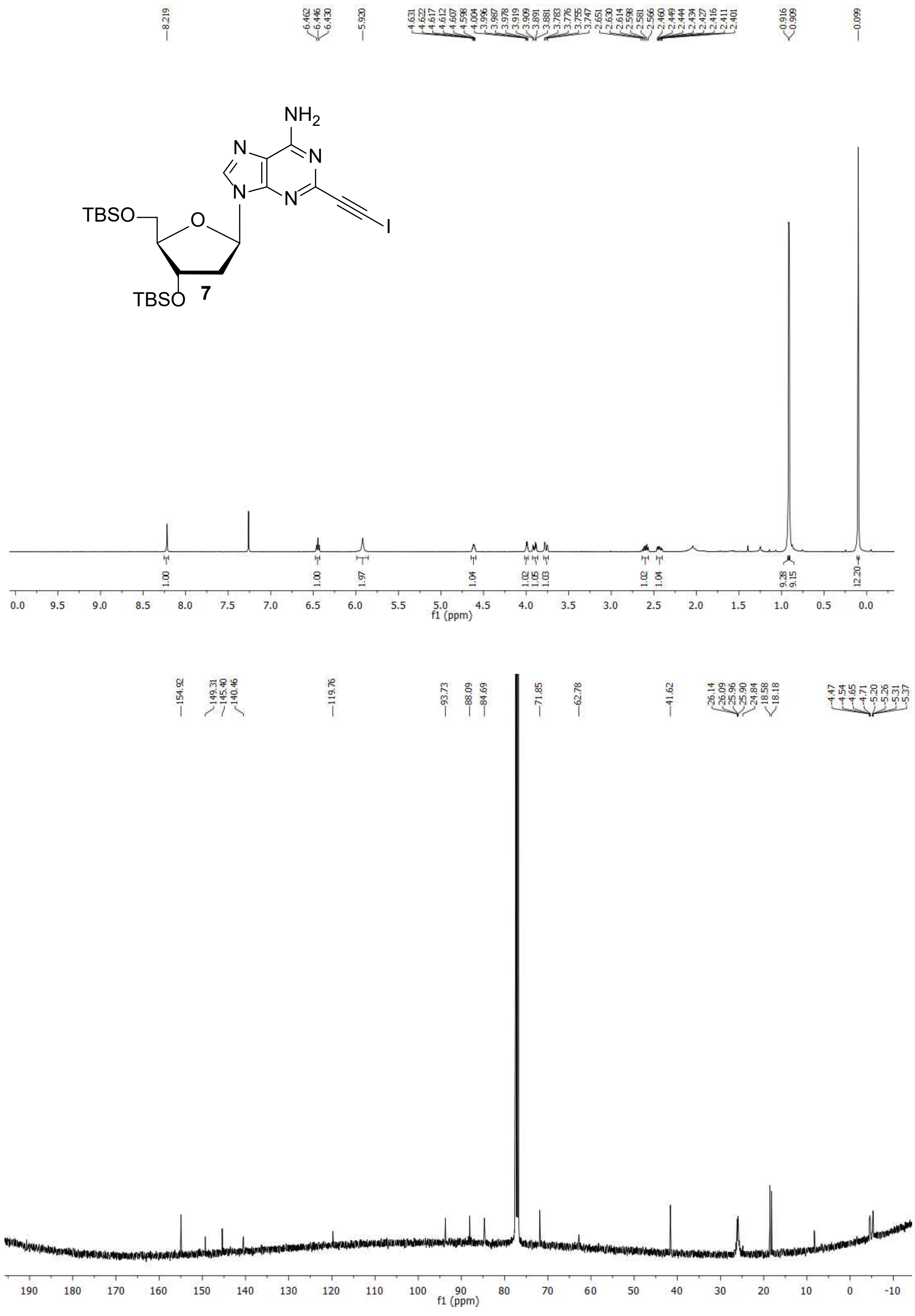

Figure S3. ^{1}H NMR and ^{13}C NMR of compound **7** in $CDCl_3$.

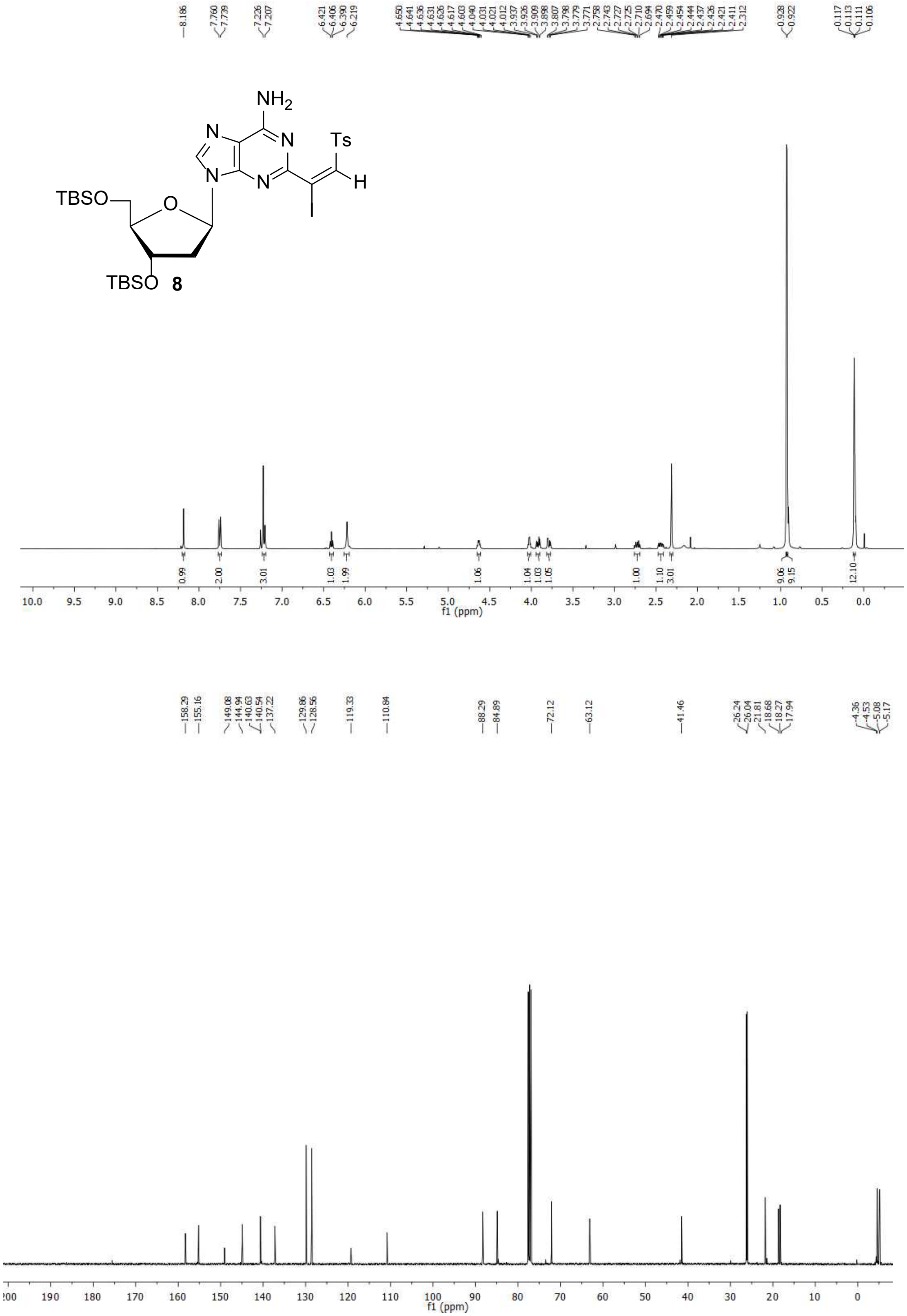

Figure S4. ^{1}H NMR and ^{13}C NMR of compound **8** in $CDCl_3$.

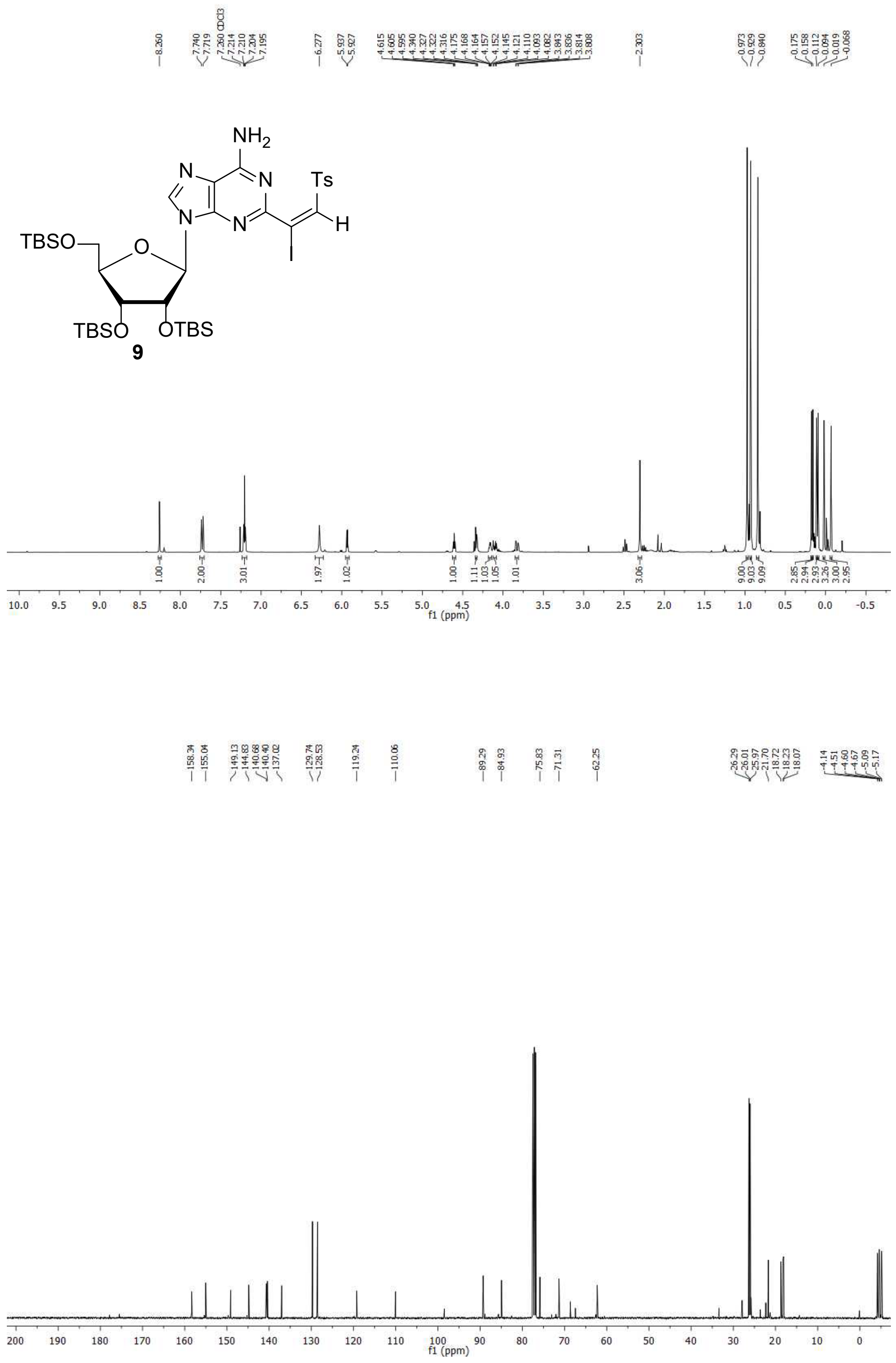

Figure S5. ^{1}H NMR and ^{13}C NMR of compound **9** in $CDCl_3$.

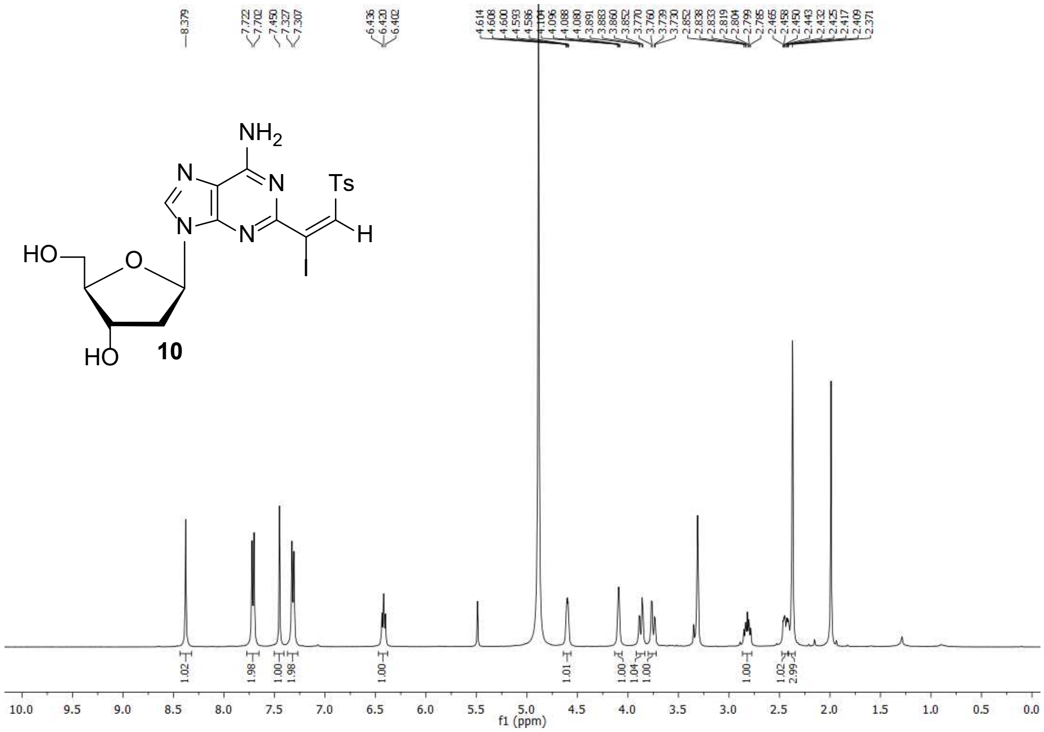

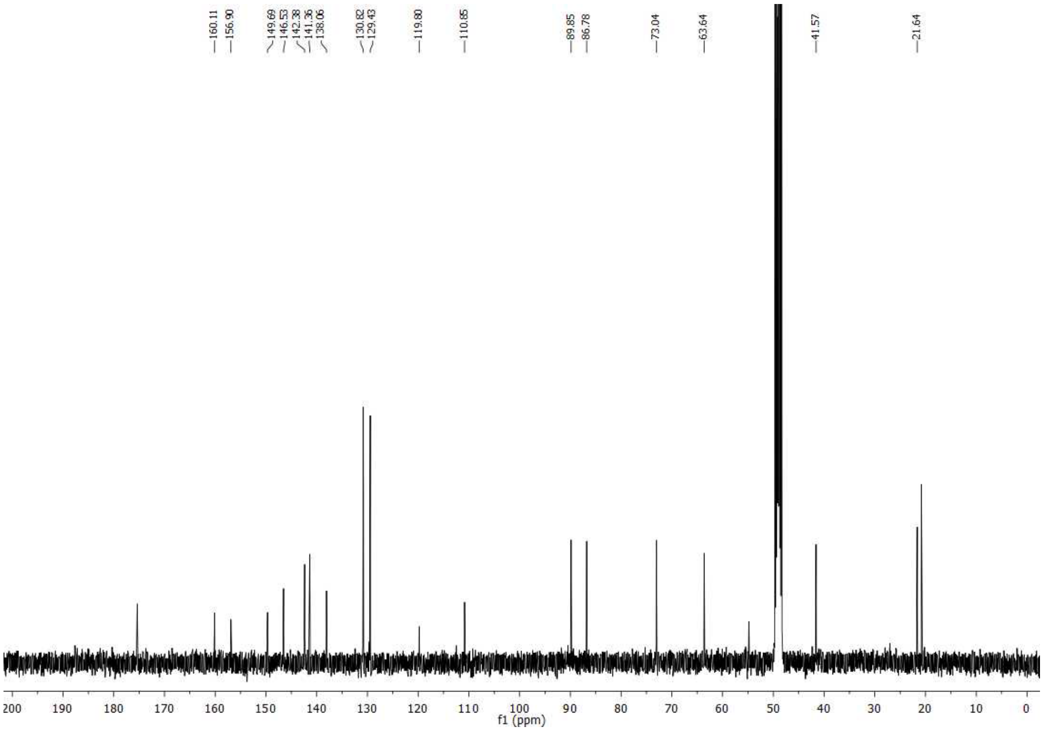

Figure S6. ^{1}H NMR and ^{13}C NMR of compound **10** in DMSO-d$_6$.

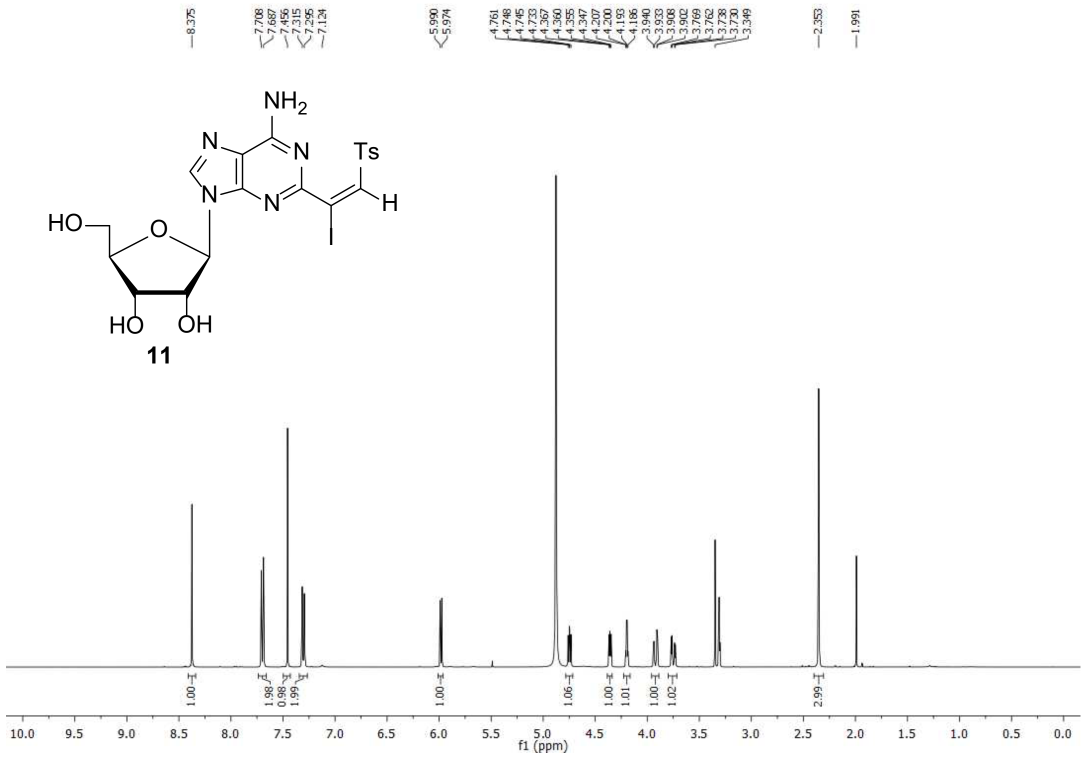

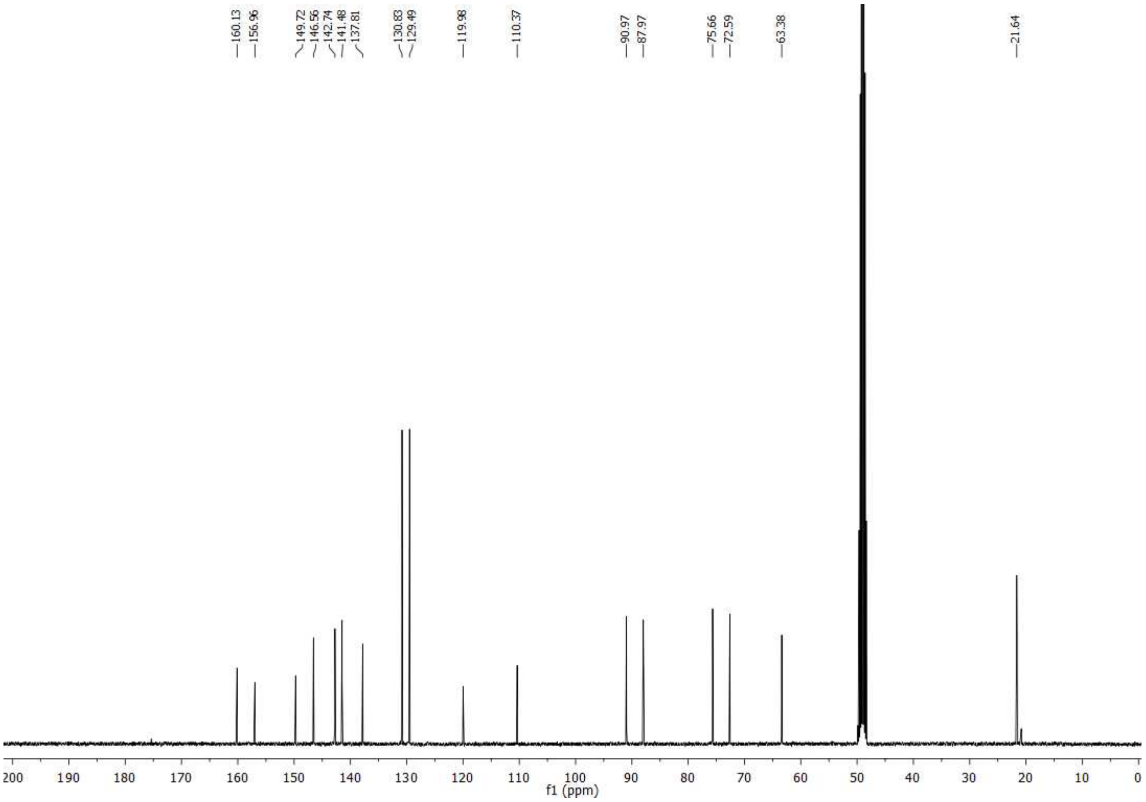

Figure S7. ^{1}H NMR and ^{13}C NMR of compound **11** in MeOH-d_4.

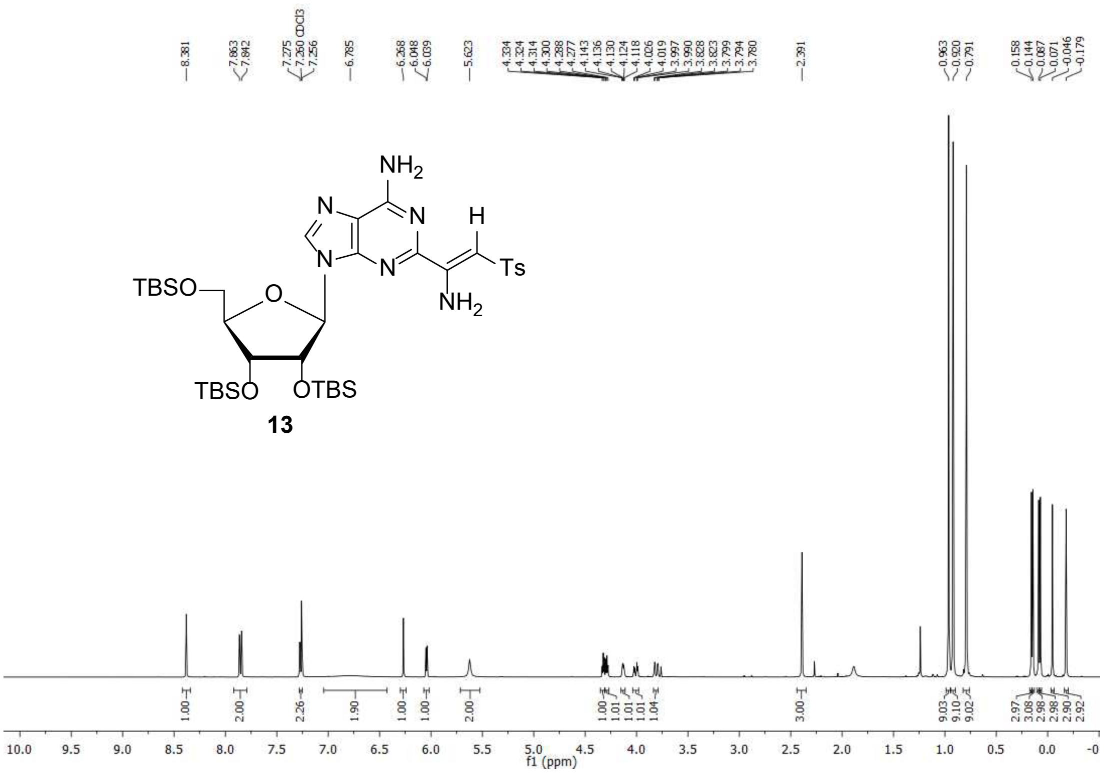

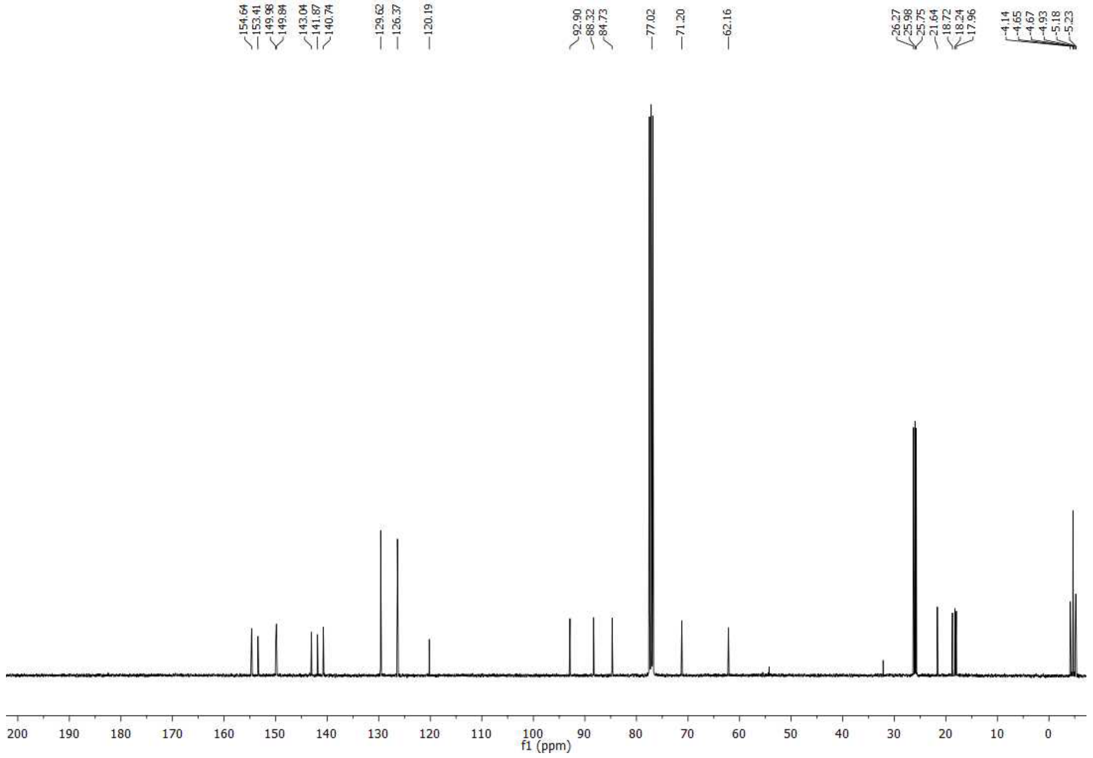

Figure S8. ^{1}H NMR and ^{13}C NMR of compound **13** in $CDCl_3$.

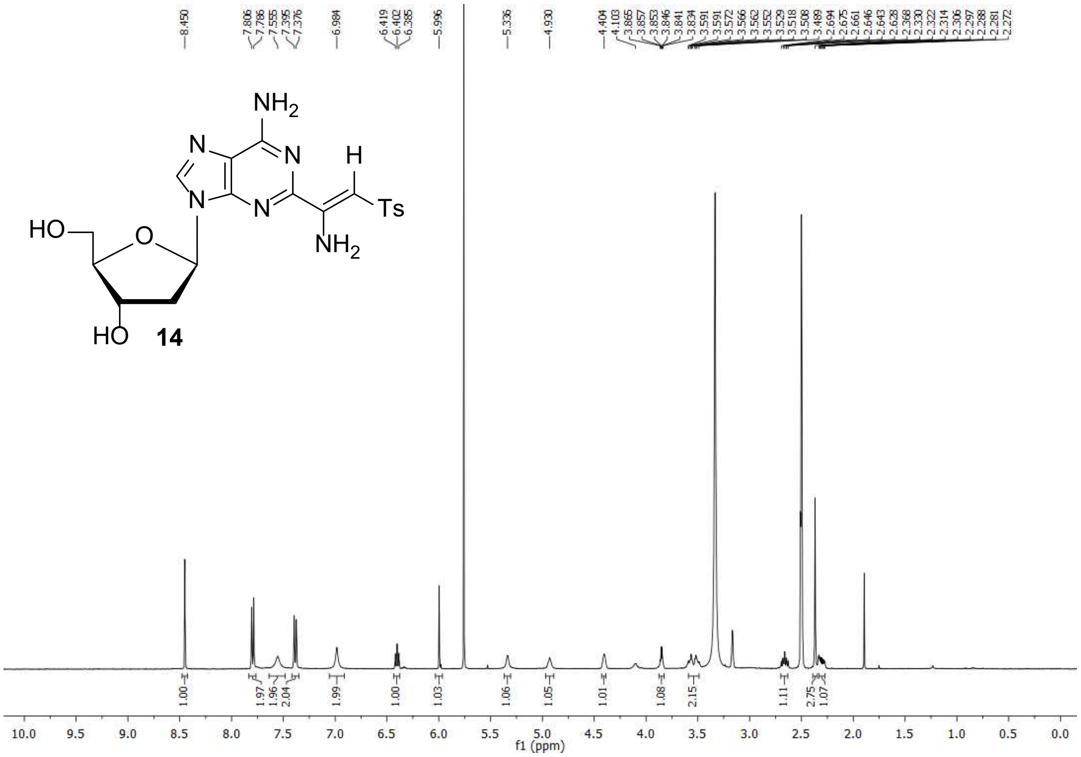

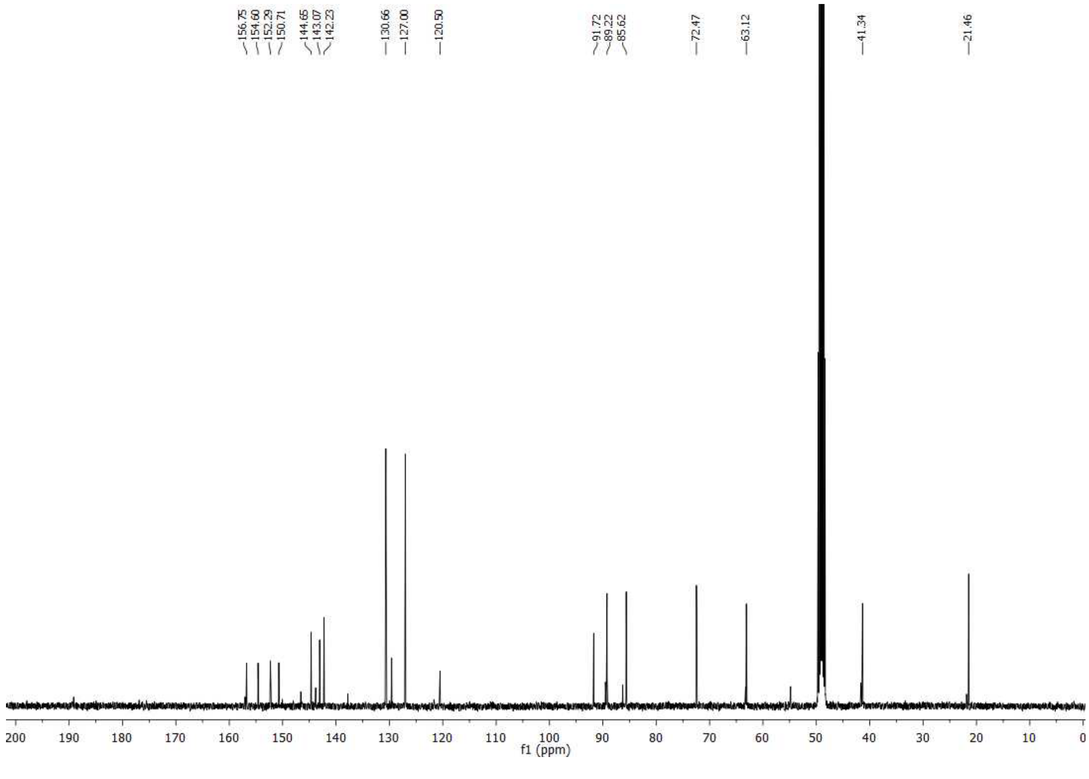

Figure S9. ^{1}H NMR and ^{13}C NMR of compound **14** in DMSO-*d*$_6$.

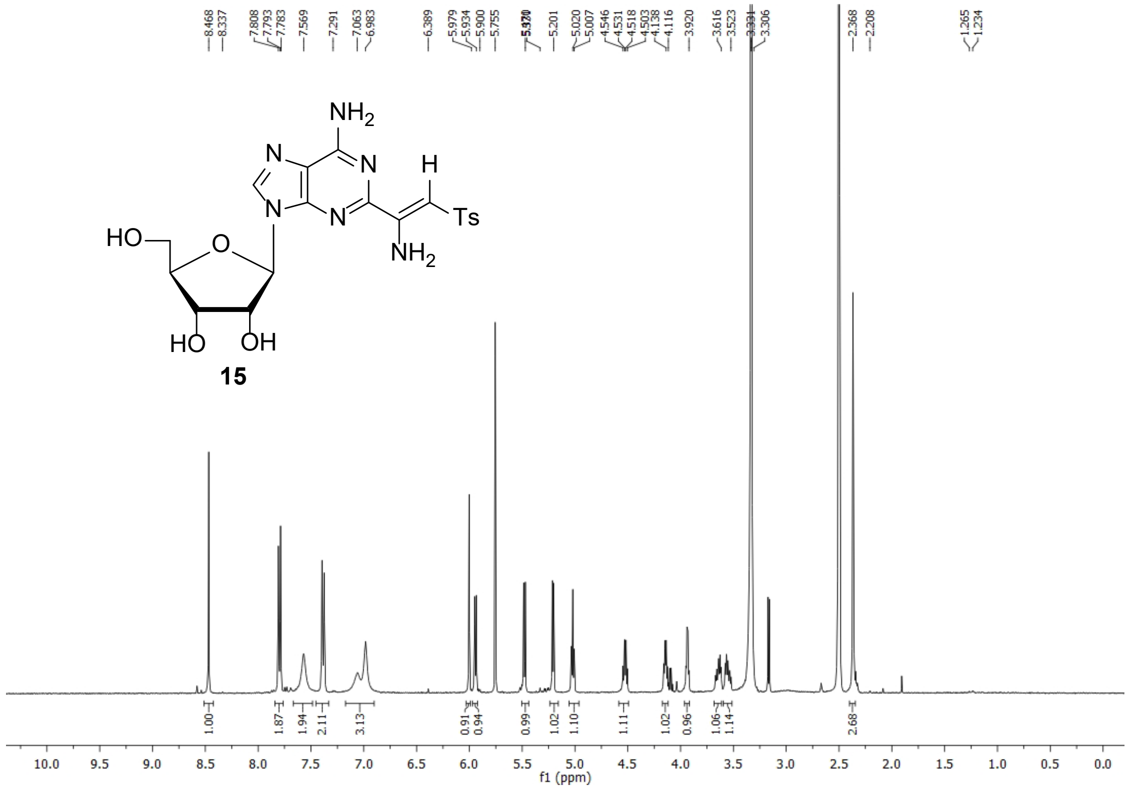

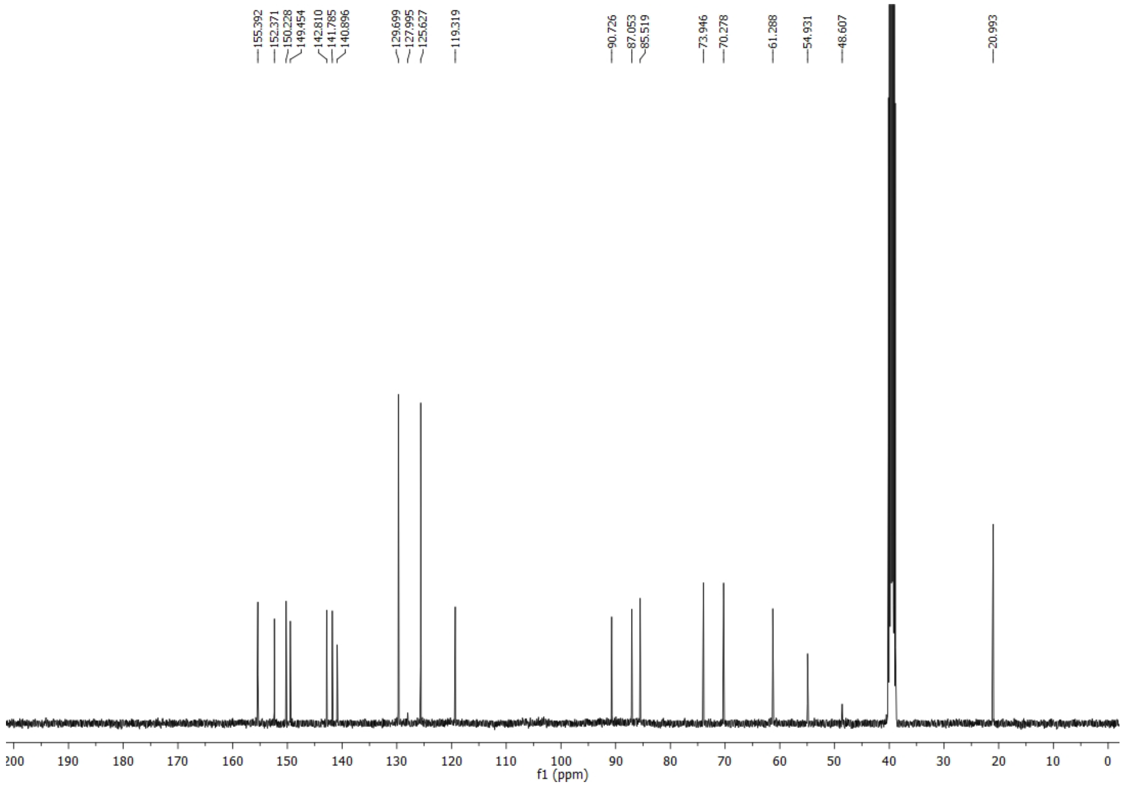

Figure S10. ^{1}H NMR and ^{13}C NMR of compound **15** in DMSO-d_6.

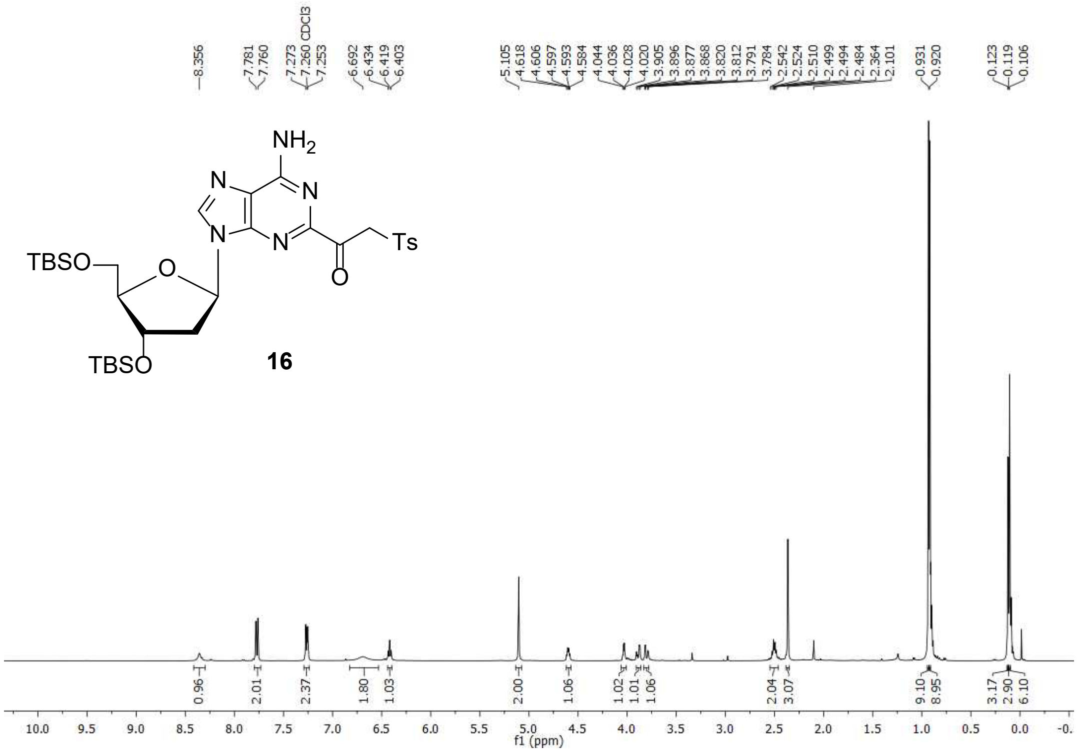

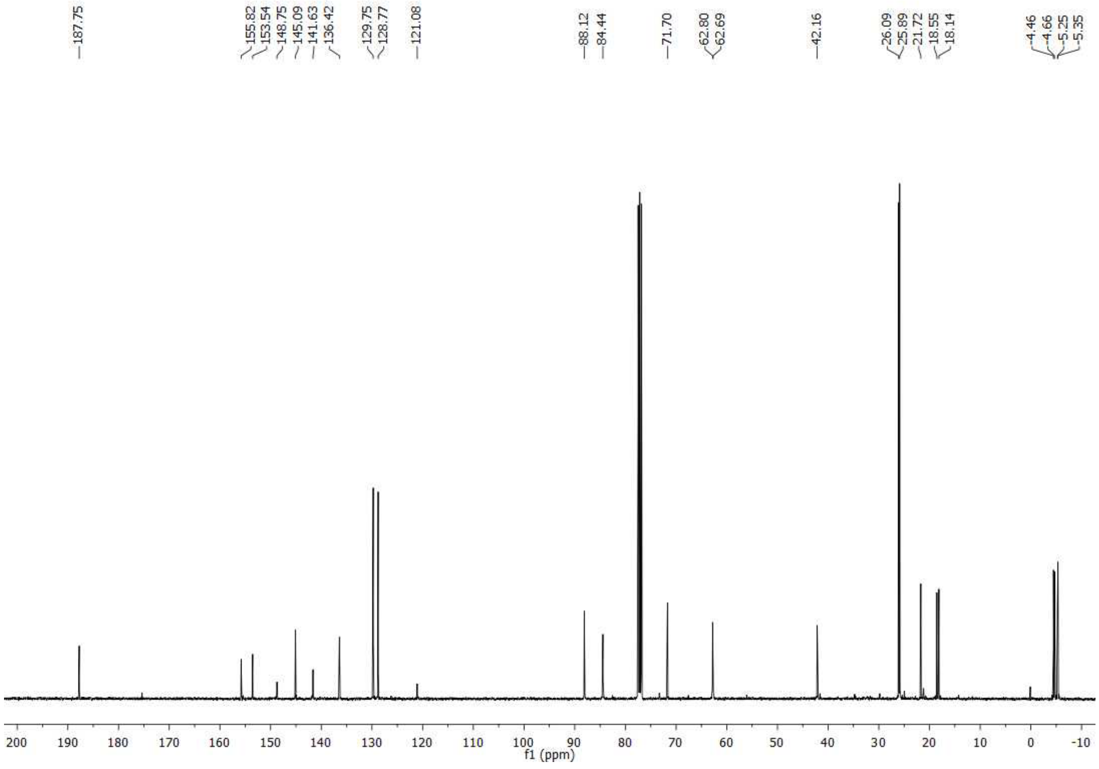

Figure S11. ^{1}H NMR and ^{13}C NMR of compound **16** in $CDCl_3$.

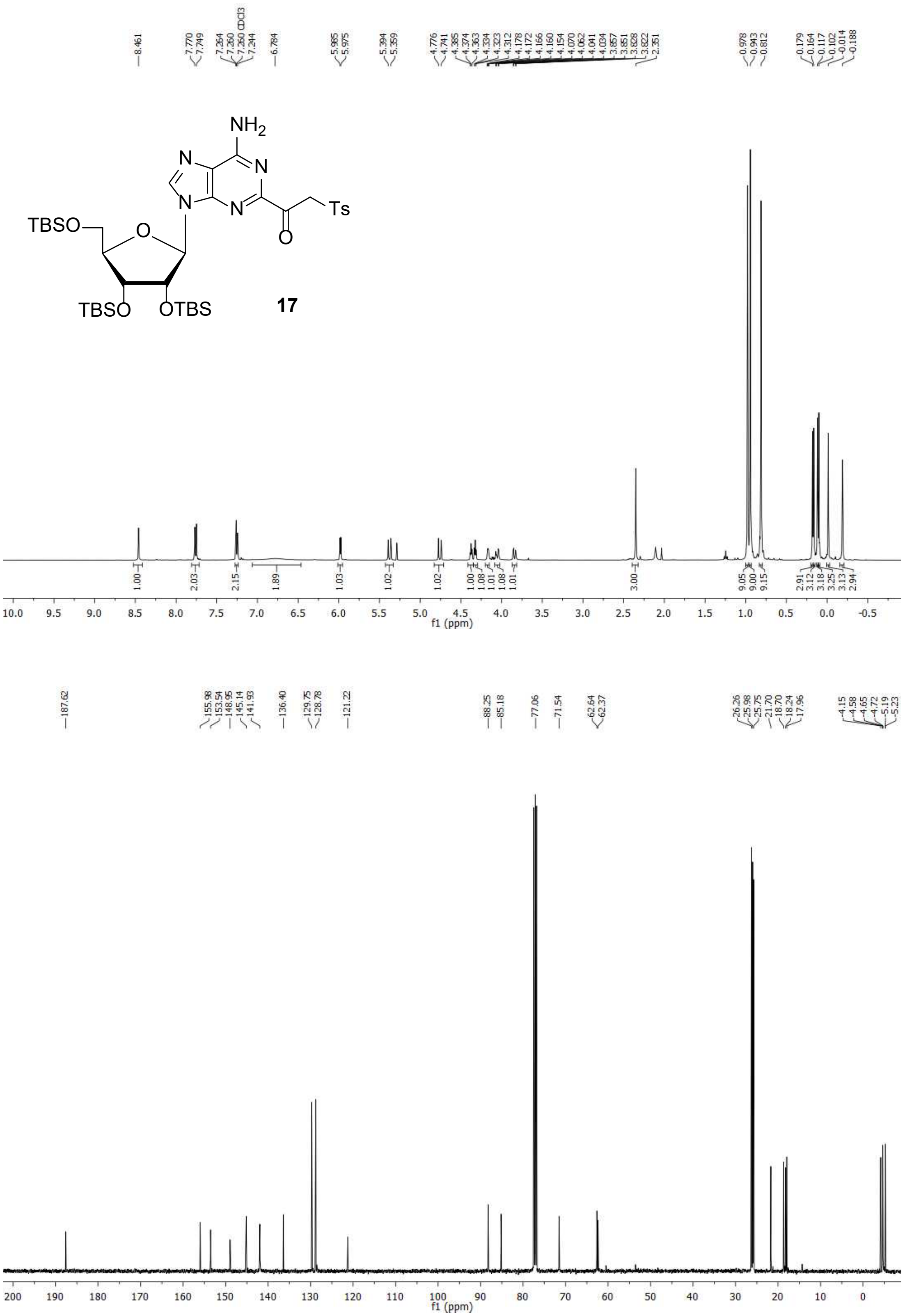

Figure S12. ^{1}H NMR and ^{13}C NMR of compound **17** in $CDCl_3$.

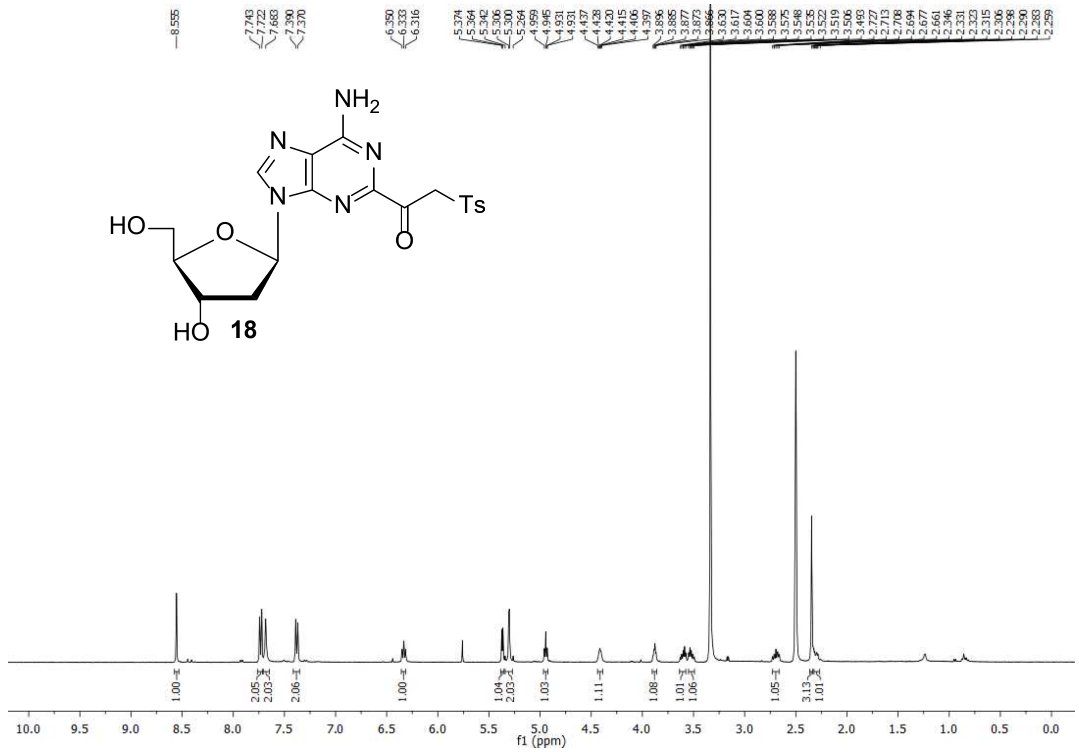

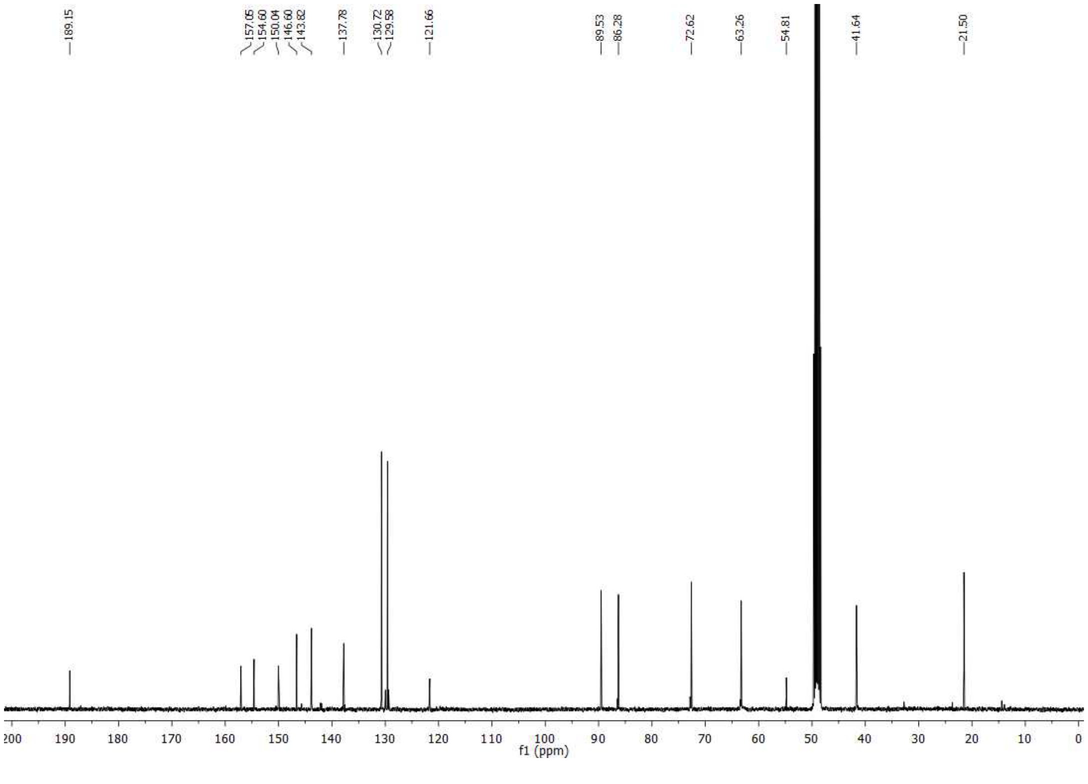

Figure S13. ^{1}H NMR and ^{13}C NMR of compound **18** in MeOH-d_4.

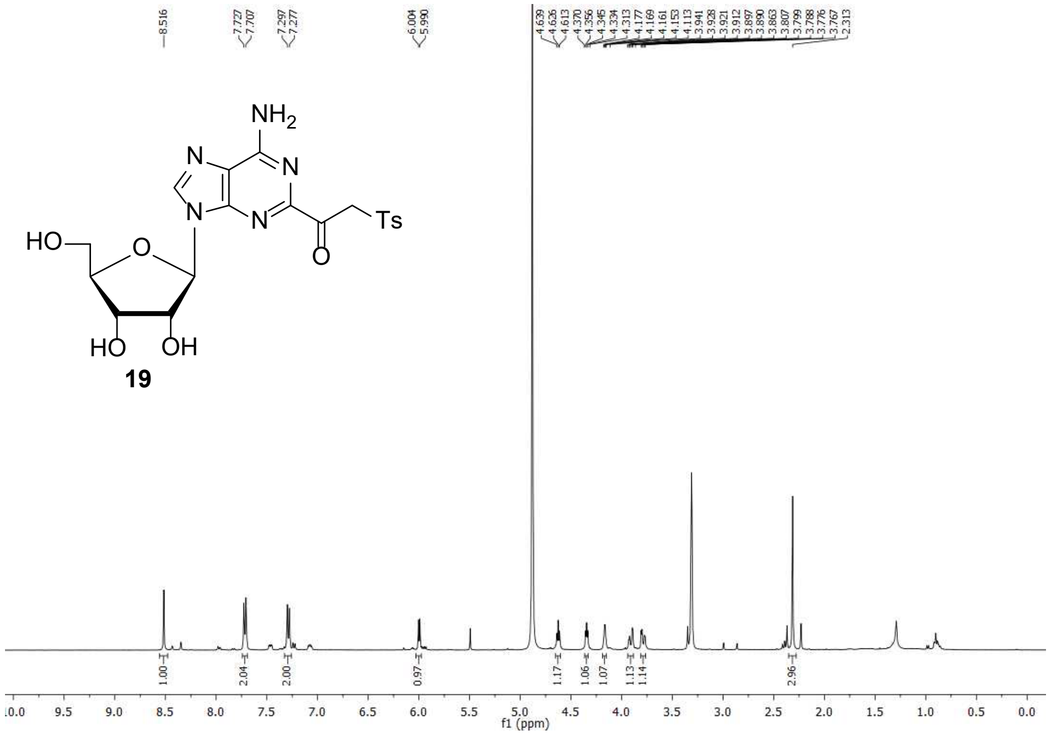

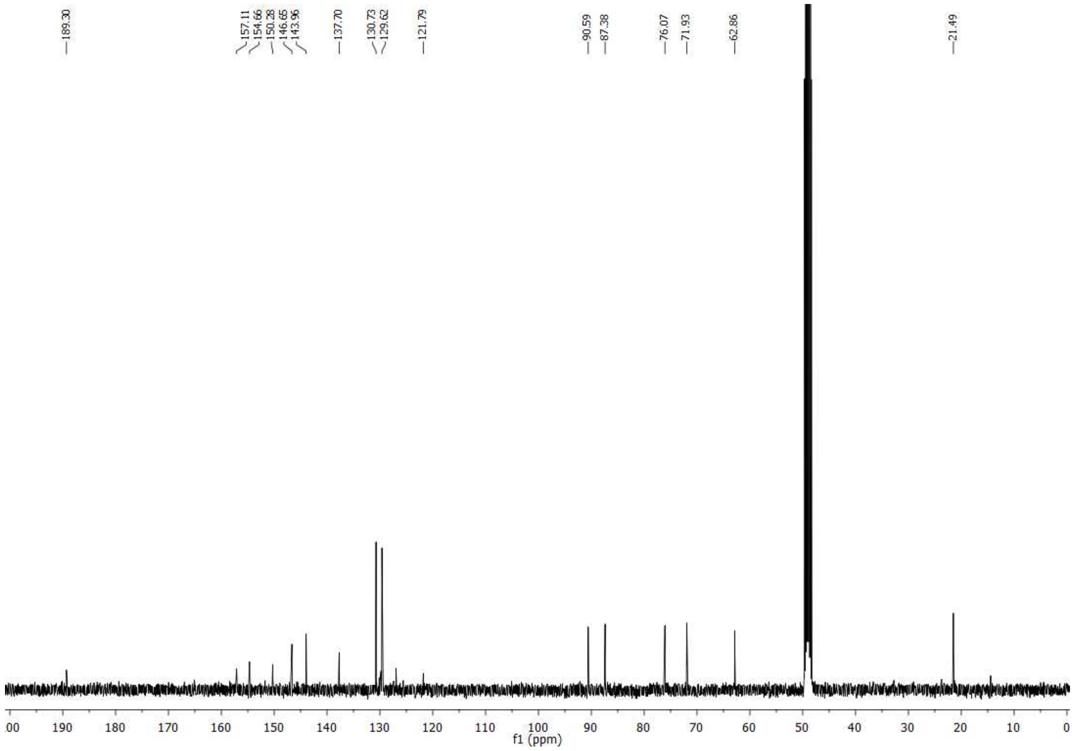

Figure S14. ^{1}H NMR and ^{13}C NMR of compound **19** in MeOH-*d*4.

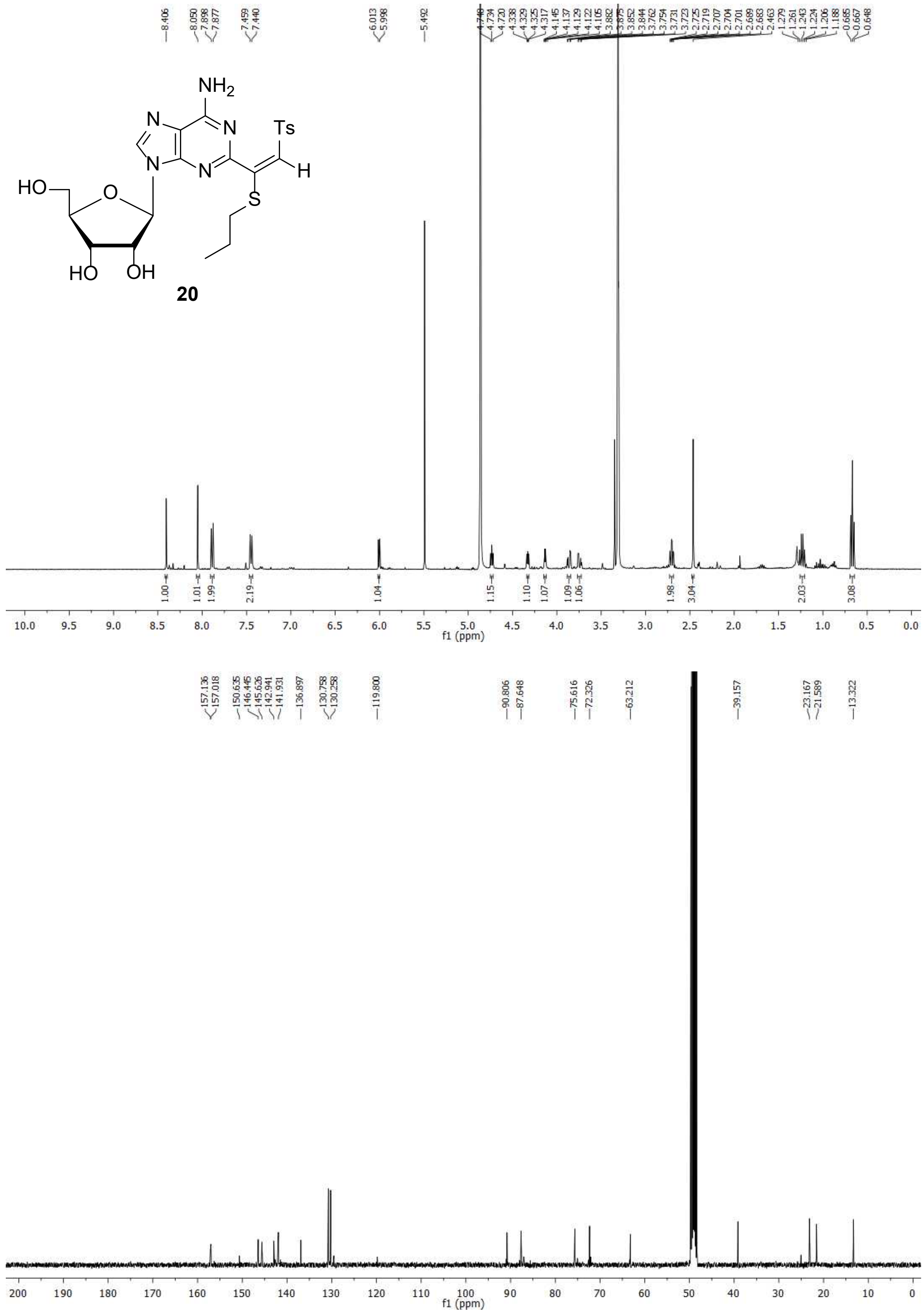

Figure S15. ^{1}H NMR and ^{13}C NMR of compound **20** in MeOH-d_4.

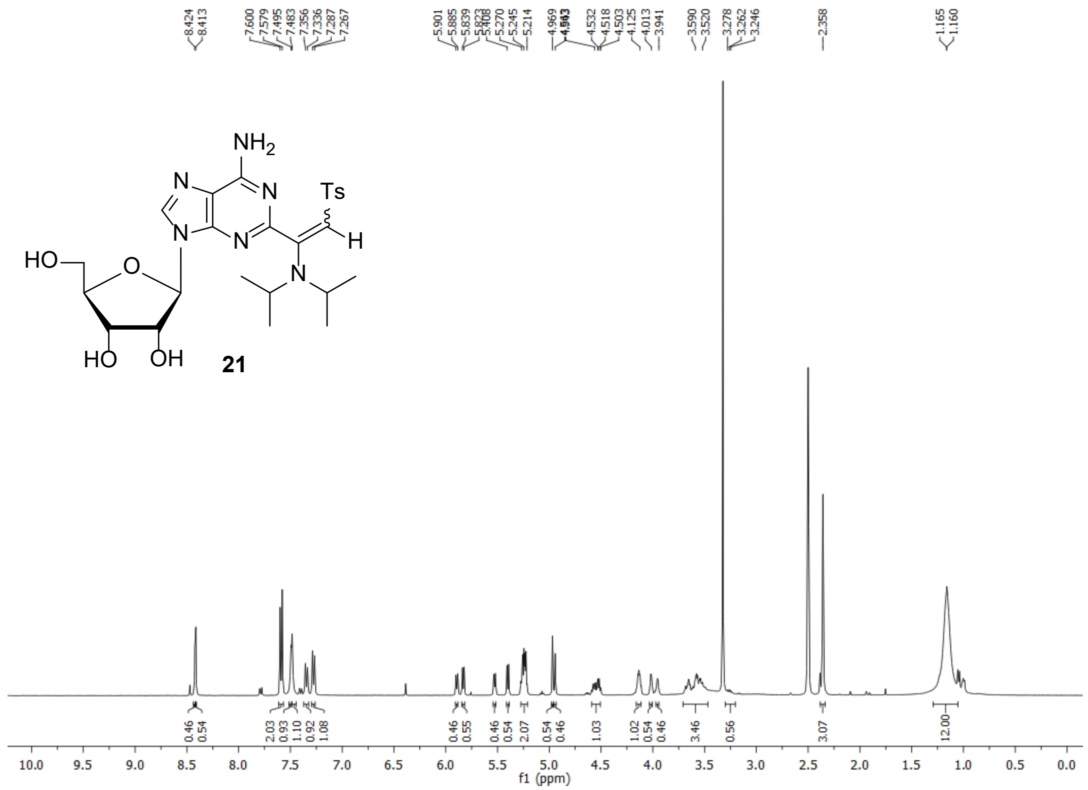

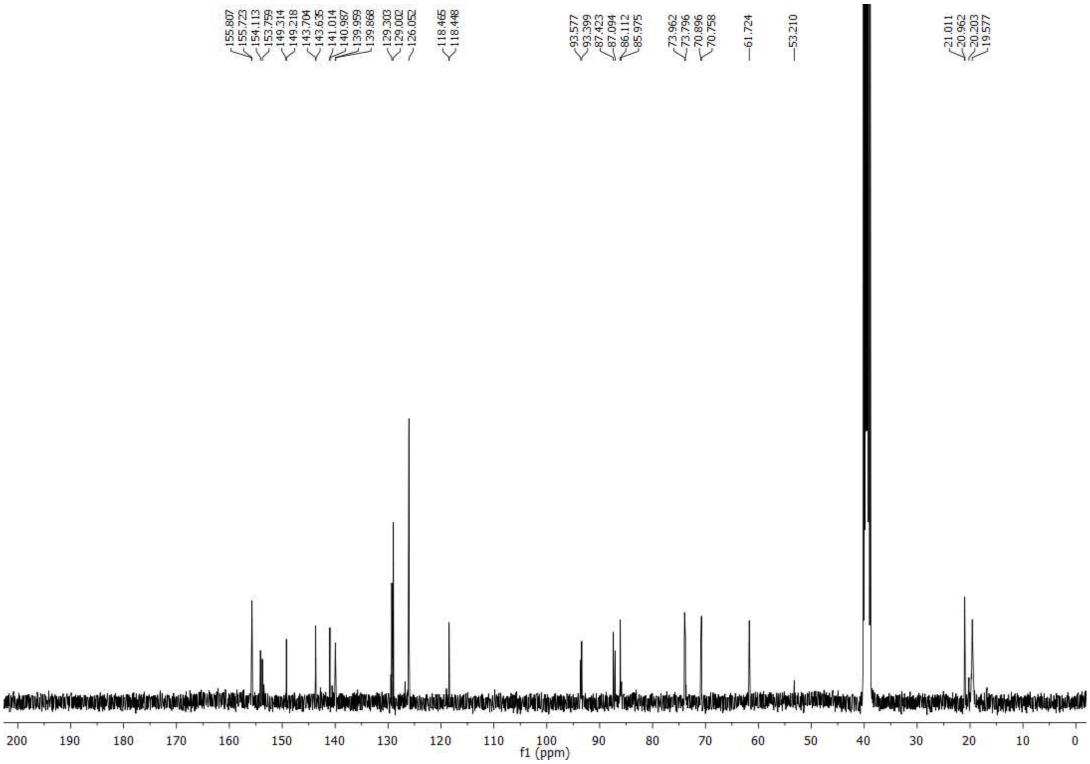

Figure S16. ^{1}H NMR and ^{13}C NMR of compound **21** in DMSO-d_6.

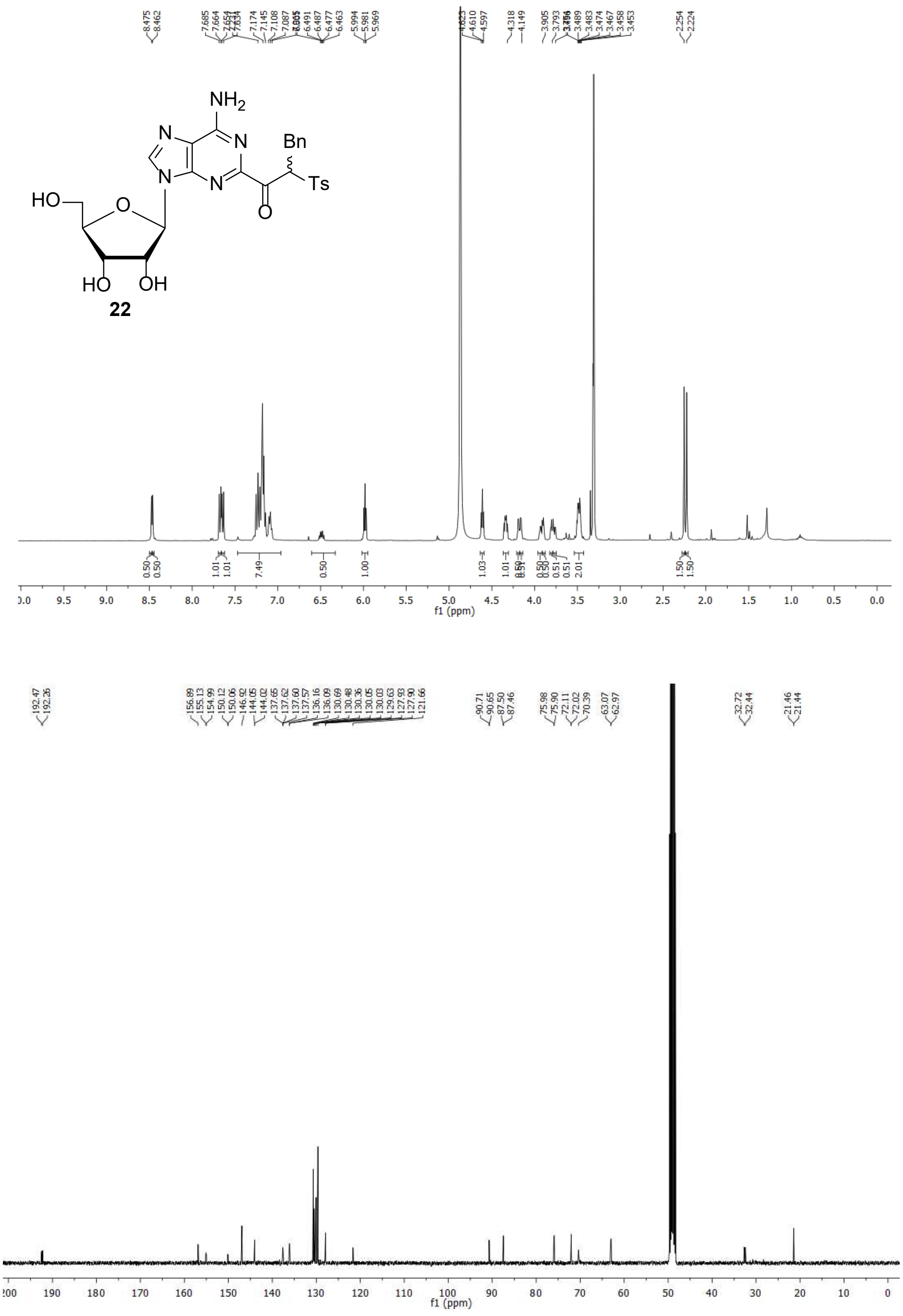

Figure S17. ^{1}H NMR and ^{13}C NMR of compound **22** in MeOH-d_4.

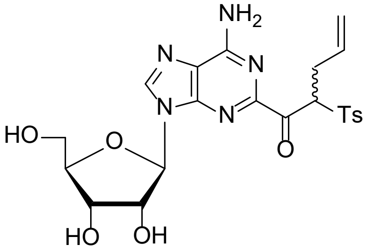

23

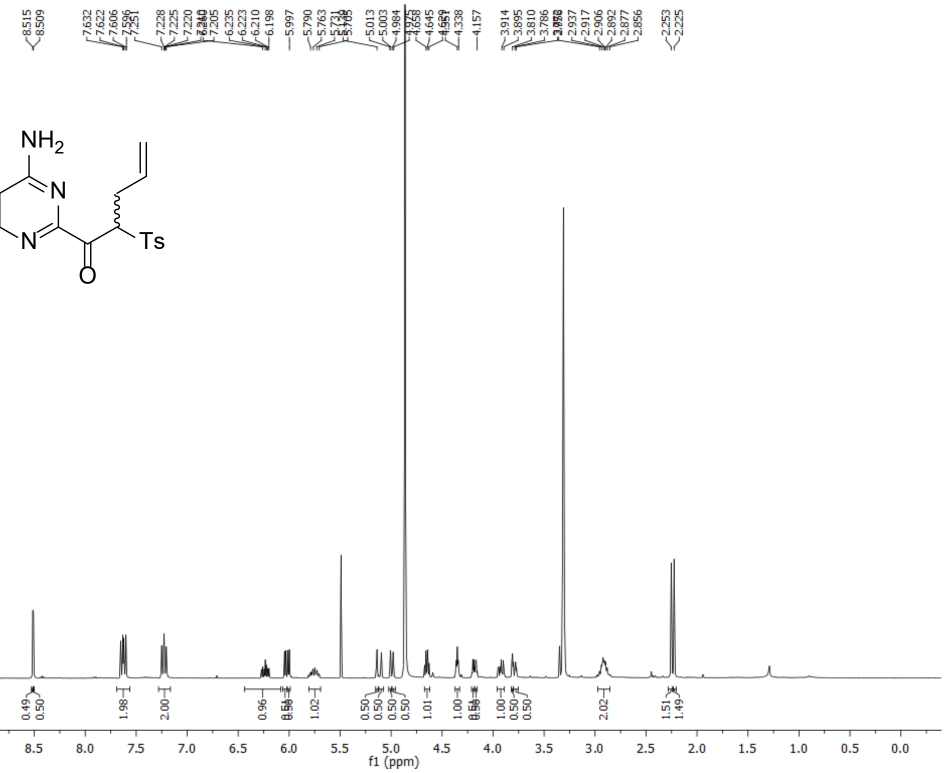

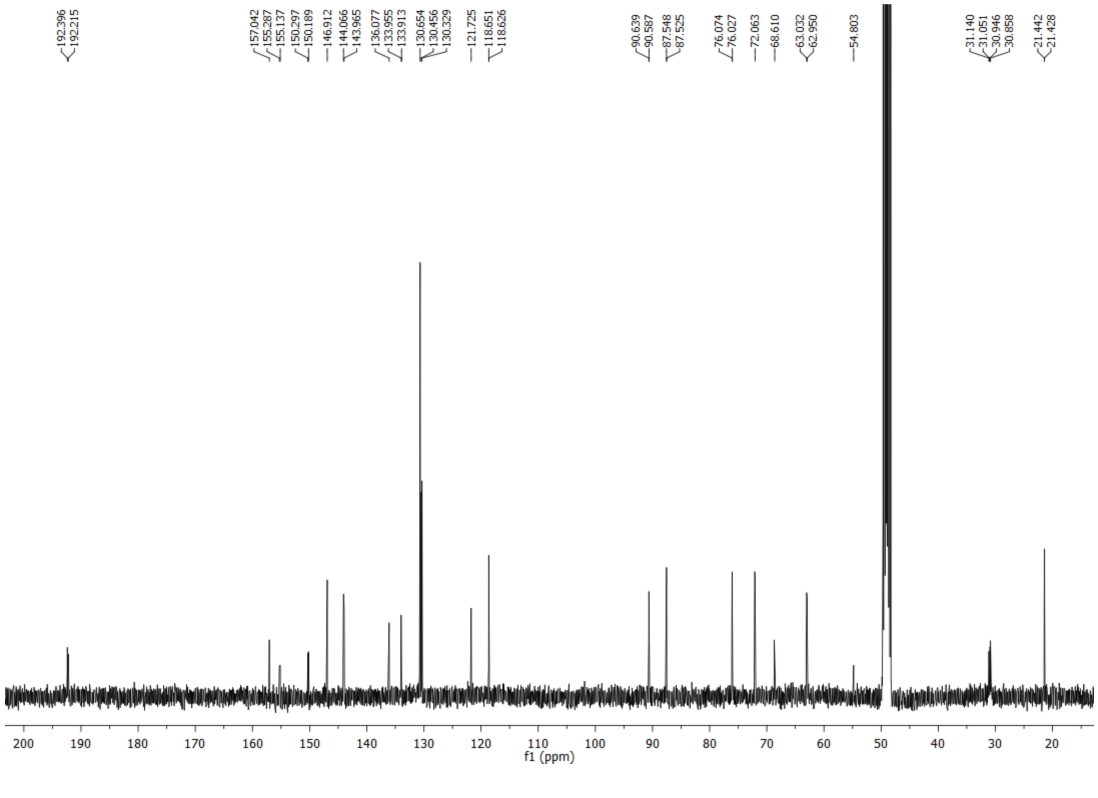

Figure S18. ^{1}H NMR and ^{13}C NMR of compound **23** in MeOH-d_4.

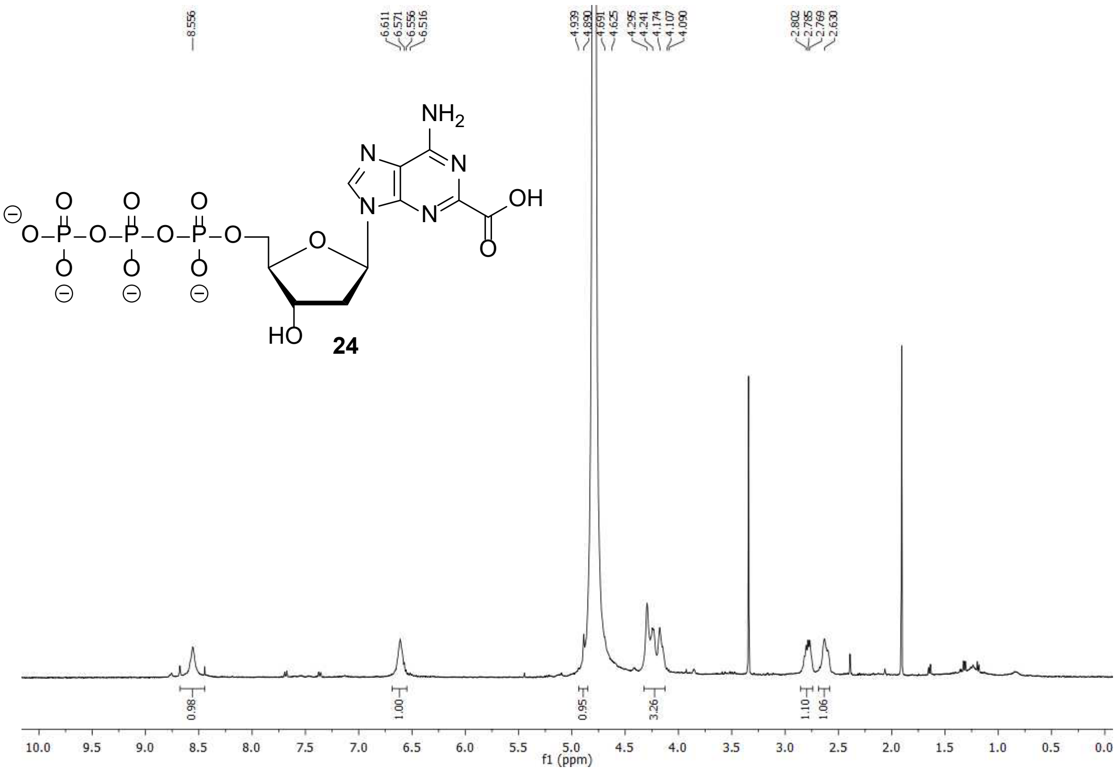

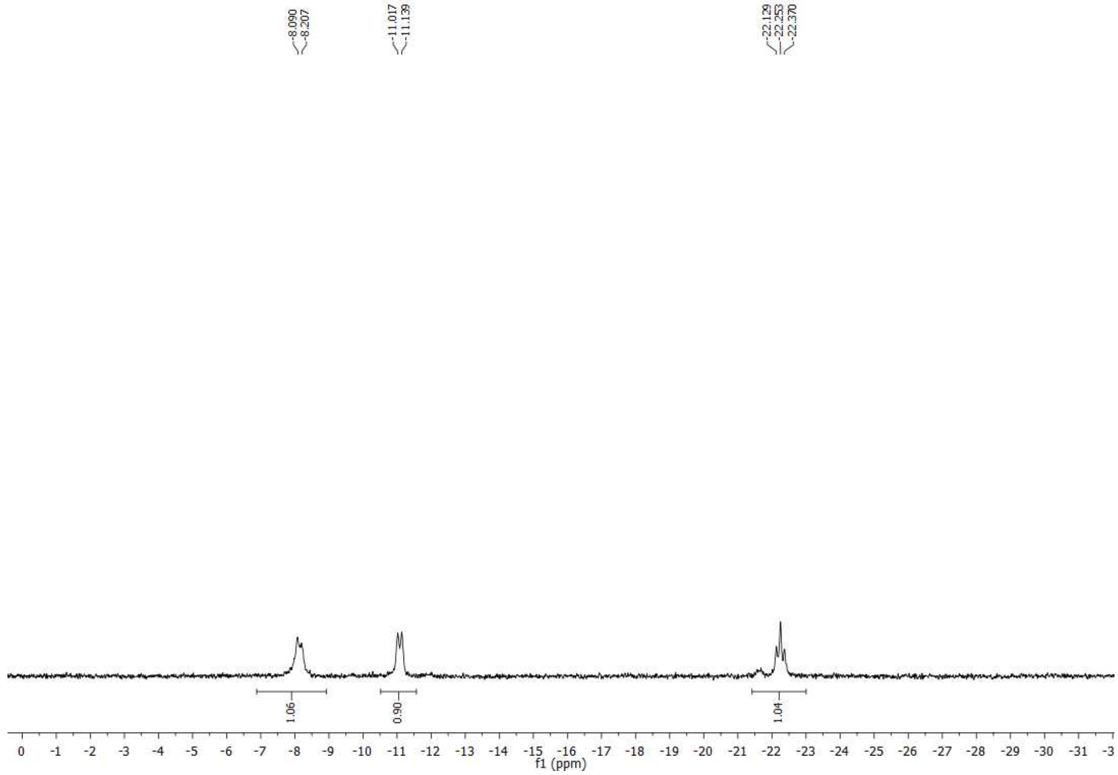

Figure S19. ^{1}H NMR and ^{31}P NMR of compound **24** in D_2O.

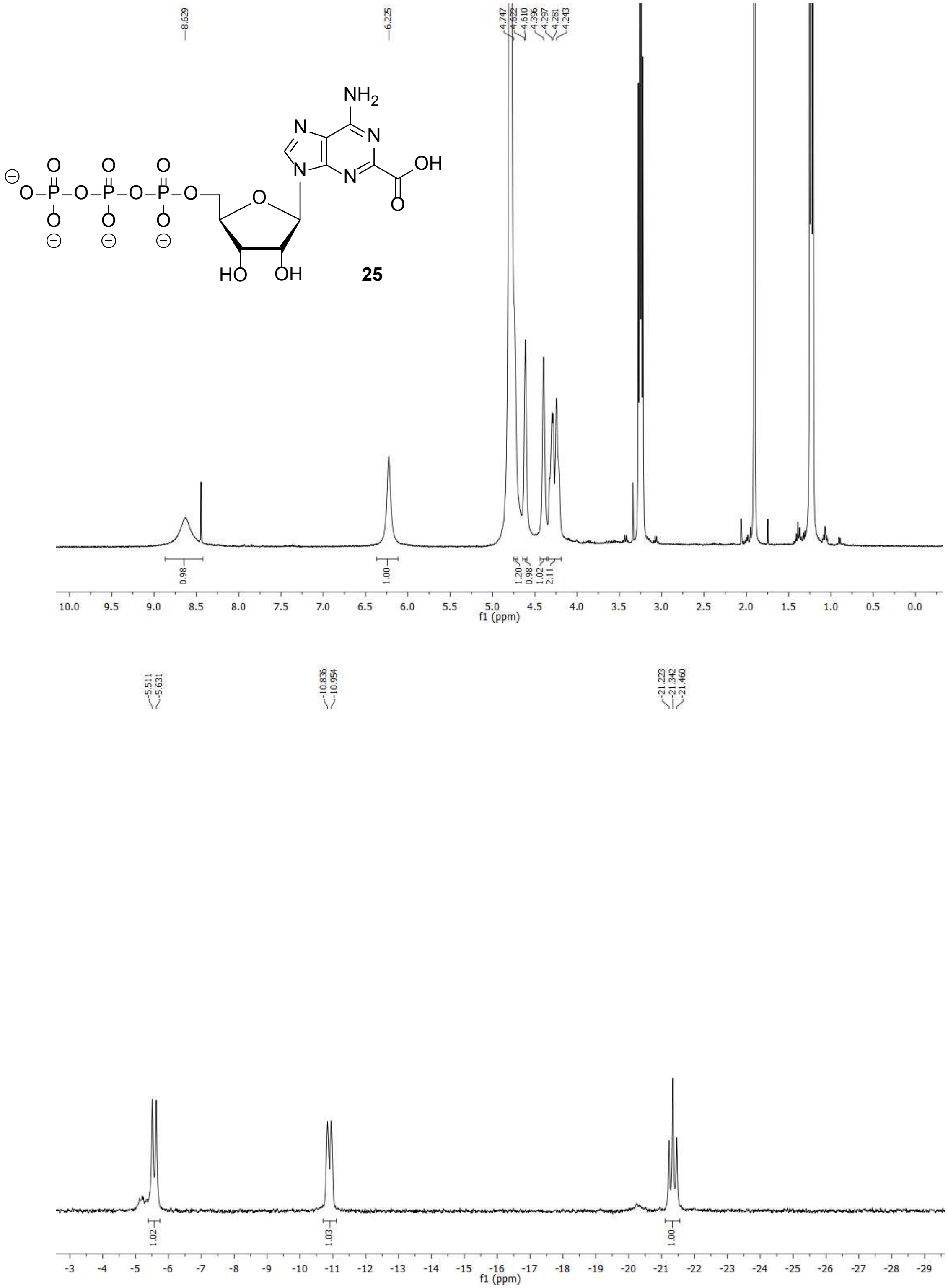

Figure S20. ^{1}H NMR and ^{31}P NMR of compound **25** in D_2O.

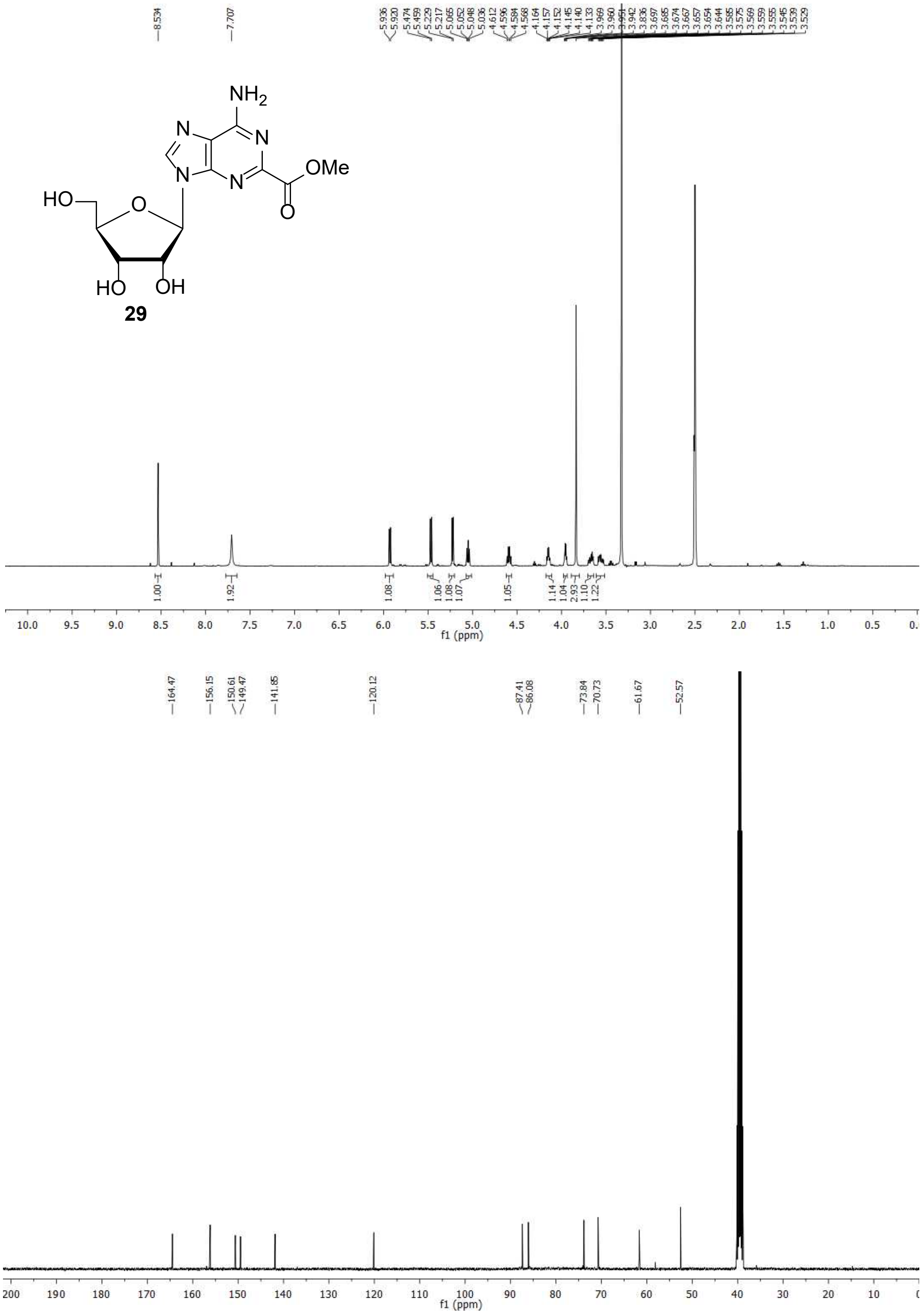

Figure S21. ^{1}H NMR and ^{13}C NMR of compound **29** in MeOH-d_4.
